# An enhancer of *Agouti* contributes to parallel evolution of cryptically colored beach mice

**DOI:** 10.1073/pnas.2202862119

**Published:** 2022-07-01

**Authors:** T. Brock Wooldridge, Andreas F. Kautt, Jean-Marc Lassance, Sade McFadden, Vera S. Domingues, Ricardo Mallarino, Hopi E. Hoekstra

**Affiliations:** ^a^Department of Organismic & Evolutionary Biology, Museum of Comparative Zoology, Harvard University, Cambridge, MA 02138;; ^b^Department of Molecular & Cellular Biology, Museum of Comparative Zoology, Harvard University, Cambridge, MA 02138;; ^c^HHMI, Harvard University, Cambridge, MA 02138;; ^d^Department of Molecular Biology, Princeton University, Princeton, NJ 08544

**Keywords:** adaptation, camouflage, convergence, deer mice, pigmentation

## Abstract

Identifying the genetic basis of repeatedly evolved traits provides a way to reconstruct their evolutionary history and ultimately investigate the predictability of evolution. Here, we focus on the oldfield mouse (*Peromyscus polionotus*), which occurs in the southeastern United States, where it exhibits considerable color variation. Dorsal coats range from dark brown in mainland mice to near white in mice inhabiting sandy beaches; this light pelage has evolved independently on Florida’s Gulf and Atlantic coasts as camouflage from predators. To facilitate genomic analyses, we first generated a chromosome-level genome assembly of *Peromyscus polionotus subgriseus*. Next, in a uniquely variable mainland population (*Peromyscus polionotus albifrons*), we scored 23 pigment traits and performed targeted resequencing in 168 mice. We find that pigment variation is strongly associated with an ∼2-kb region ∼5 kb upstream of the *Agouti signaling protein* coding region. Using a reporter-gene assay, we demonstrate that this regulatory region contains an enhancer that drives expression in the dermis of mouse embryos during the establishment of pigment prepatterns. Moreover, extended tracts of homozygosity in this *Agouti* region indicate that the light allele experienced recent and strong positive selection. Notably, this same light allele appears fixed in both Gulf and Atlantic coast beach mice, despite these populations being separated by >1,000 km. Together, our results suggest that this identified *Agouti* enhancer allele has been maintained in mainland populations as standing genetic variation and from there, has spread to and been selected in two independent beach mouse lineages, thereby facilitating their rapid and parallel evolution.

## Significance

Oldfield mice have independently colonized the white-sand beaches of Florida’s Gulf and Atlantic coasts, where they have evolved light fur that camouflages them from visually hunting predators. We find that fur color is strongly associated with DNA variation in a small regulatory region of the *Agouti signaling protein*, which contains an enhancer that drives expression in mouse skin. This regulatory allele is found in all light-colored beach mice on both coasts, despite being separated by >1,000 km. Based on patterns of DNA variation within and between populations, our results suggest that this *Agouti* allele arose in the mainland and then, has spread to and been selected in two independent beach mouse lineages, thereby facilitating their rapid and parallel evolution.

To gain a complete picture of adaptation, we strive to understand both the molecular mechanisms and the evolutionary processes underlying trait evolution. On one hand, identifying the molecular basis of phenotypic adaptation can provide an opportunity to learn how traits vary—in particular, how specific changes in DNA can affect protein function or expression during development to produce the trait of interest. On the other hand, the evolutionary history of a specific allele can provide insights into when and why traits evolve. Importantly, an allele may be influenced by a combination of neutral and selective forces, which together explain its current distribution and frequency. Thus, the identification of a causal gene or better, a small gene region or mutation can serve as a handle with which to probe both the proximate (how) and ultimate (when/why) drivers of trait variation.

Cases of repeated evolution provide a particularly appealing context for understanding the drivers of adaptation. For example, one can ask the following question. Did similar phenotypes arise via the same or different molecular changes? While there are empirical examples of selection from new mutations (1–3), it has been suggested that rapid adaptation, in particular within species, may be fueled by selection on the same alleles from preexisting genetic variation (refs. 4–6; reviewed in ref. 7). Moreover, it has been argued that changes in *cis*-regulatory elements may be the primary substrate of adaptation (8–10), although many examples of protein-coding changes (refs. 11–13; reviewed in ref. 14) or combinations of both regulatory and coding changes (15) have been identified. Nonetheless, when regulatory change has been implicated in repeated evolution, it is still rare that the causal regions, elements, or mutations have been identified (1, 16). This is in part due to the complexity of gene regulatory landscapes and the relative difficulty in testing the effects of a noncoding allele (17). By contrast, coding mutations are generally more amenable to identification and functional validation; therefore, when precise mutations have been shown to drive repeated evolution across species, they most commonly correspond to coding mutations (18). Thus, it remains difficult to determine the extent to which similar or different mutations contribute to repeated phenotypic evolution and where in the genome they occur.

Variation in pigmentation has long served as a model for the study of adaptation. At the molecular level, the genes and pathways involved in vertebrate pigmentation have been well characterized (19). At the phenotypic level, color can vary dramatically in the wild, be measured straightforwardly, and have clear links to fitness (20). One classic example of repeated color evolution involves beach mice in the southeastern United States. Beach mouse subspecies on Florida’s Gulf and Atlantic coasts have independently evolved light coloration from a dark-colored mainland ancestor (21). Previous work identified three genomic regions involved in differences between Gulf coast beach mice and mainland mice (22), in which two pigment genes have thus far been implicated: the *Melanocortin-1 receptor* [*Mc1r* (19)] and the *Agouti signaling protein* [*ASIP* (23)]. The interaction between *Mc1r* and *Agouti* mediates the switch from dark (eumelanin) to light pigment (pheomelanin) production in mammals (24–26).

In Gulf coast beach mice, a single missense mutation in *Mc1r* reduces the receptor’s signaling ability, thereby contributing to light pigmentation in some (but not all) populations (19), but there are no differences in *Mc1r* expression level (22). By contrast, differences in *Agouti* transcript abundance are associated with pigment variation, with higher expression in Gulf coast beach mice than mainland mice, but there are no differences in the *Agouti* coding sequence (22). Therefore, while changes in genes at multiple levels of the pigment pathway have been implicated in the evolution of camouflaging coloration in Gulf coast beach mice (i.e., *Mc1r* and *Agouti*), we have an incomplete understanding of the regulatory mechanism driving differences in *Agouti* expression.

The genes (and mutations) contributing to the light coats of the Atlantic coast beach mice have remained even more elusive. For example, the *Mc1r* amino acid change found in Gulf coast mice is absent from Atlantic coast mice (19), and no new *Mc1r* mutations are associated with color variation or have a measurable effect on *Mc1r* function (21). Moreover, there are no differences in the *Agouti* coding region between mainland and Atlantic coast beach mice (22), and changes in *Agouti* expression have not been measured. Without knowledge of the genes or mutations underlying light pigmentation in the Atlantic coast beach mice, an outstanding question remains: Is the remarkable similarity in coloration between Gulf and Atlantic coast beach mice due to the same or distinct pigmentation alleles?

Here, we return to the classic case of adaptation in Gulf and Atlantic coast beach mice, first described over a century ago (27, 28), and capitalize on naturally occurring color variation in a single mainland population to identify the molecular basis of adaptation by first generating a chromosome-level genome assembly for *Peromyscus polionotus*. Next, we employ an association-mapping approach to identify an ∼2-kb previously uncharacterized noncoding region of *Agouti* associated with color variation. We then show that this 2-kb region can drive dermal expression in *Mus* embryos, demonstrating its regulatory activity in the skin during the establishment of pigmentation. Finally, we reveal the evolutionary history of this regulatory element to show both strong selection on the light *Agouti* allele in a phenotypically variable population and that this same allele is fixed in beach mice of both the Gulf and Atlantic coasts. Together, we find that both the molecular basis and evolutionary history differ markedly between two key genes involved in beach mouse adaptation—*Agouti* and *Mc1r*—highlighting that there can be multiple genetic solutions to the same ecological challenge, even within species.

## Results

### Assembly of a High-Quality Chromosome-Level Genome for *P. polionotus*.

We first generated whole-genome sequencing (WGS) data and assembled a de novo high-quality reference genome for the oldfield mouse, *Peromyscus polionotus subgriseus* (BioProject no. PRJNA494229). The final genome was 2.645 Gb in length with an N50 scaffold length of 13 Mb. We could anchor 97% of the de novo assembled bases into 23 autosomes and the X chromosome using high-density genetic linkage maps for *Peromyscus*. Our estimates indicate that the assembly contains 95.4 and 94.8% of single-copy core mammalian and euarchontoglire genes, respectively. Our annotation strategy, which combined comparative in silico and evidence-based approaches, identified 18,502 protein-coding genes having orthologs in the *Mus* genome, 536 paralogs of *Mus* genes, and 1,912 additional genes showing homology with known proteins from curated databases. This high-quality genome enables evolutionary analyses of genome-wide variation across populations of this species.

### Recent and Independent Evolution of Beach Mice on the Gulf and Atlantic Coasts.

**Fig. 1.**
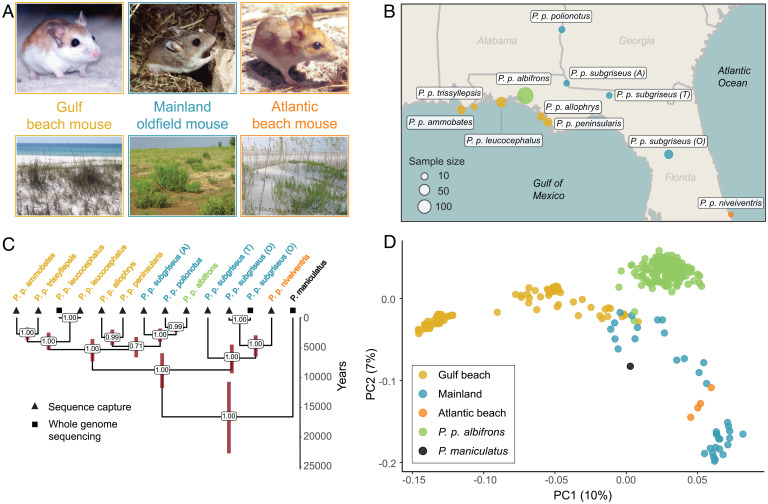
Distribution and relationships of beach and mainland subspecies of *P. polionotus*. (*A*) Representative images of beach and mainland subspecies of *P. polionotus* as well as corresponding habitats. (*B*) Map of the southeastern United States showing sampling locations of populations included in this study (*SI Appendix*, Table S1 has details). Sample sizes are indicated in parentheses; the area of each circle corresponds to sample size. *P. p. subgriseus* was sampled from three locations: A, Apalachee; O, Ocala; T, Tall Timbers Research Station. (*C*) Time-scaled phylogeny of sampled populations. Numbers at nodes represent bootstrap support; red bars are 95% CIs of divergence time. Populations are annotated with one of two sequencing strategies used in this study. (*D*) The first two dimensions of a PCA based on genotype probabilities; each dot represents an individual with sample sizes given in *B*.

To better estimate the timing and pattern of divergence in the beach and mainland subspecies (Fig. 1*A*), we sampled six beach and five mainland populations, all together representing 9 of the 14 extant *P. polionotus* subspecies (Fig. 1*B*) as well as the closely related sister species, *Peromyscus maniculatus nubiterrae*. Using 1,000 randomly distributed genome-wide single nucleotide polymorphisms (SNPs) derived from putatively neutral regions in a targeted sequence-capture dataset, we generated a highly supported phylogeny confirming the independent origin of beach mice on the Gulf and Atlantic coasts from an ancestral mainland form (Fig. 1*C*), consistent with previous studies (21, 29–31). The Gulf coast beach mice form a paraphyletic group with adjacent mainland populations, all of which share a common ancestor between 3.5 and 7.2 thousand y ago (kya). Similarly, the Atlantic coast beach mice share a common ancestor with their closest mainland counterparts 2.9 to 6.4 kya, suggesting that both Gulf and Atlantic beach lineages originated at approximately the same time. In general, we find that the relationships of subspecies in the phylogeny mirror their geographic distribution, a pattern that is supported by a genetic Principal Components Analysis (gPCA) based on genotype likelihoods from all loci in the sequence-capture dataset (Fig. 1*D*). The evolutionary history of both Gulf and Atlantic beach mice as well as several mainland populations provides a demographic context in which to understand the evolution of crypsis.

### Phenotypic Variation in a Single Mainland Population (*Peromyscus polionotus albifrons*).

**Fig. 2.**
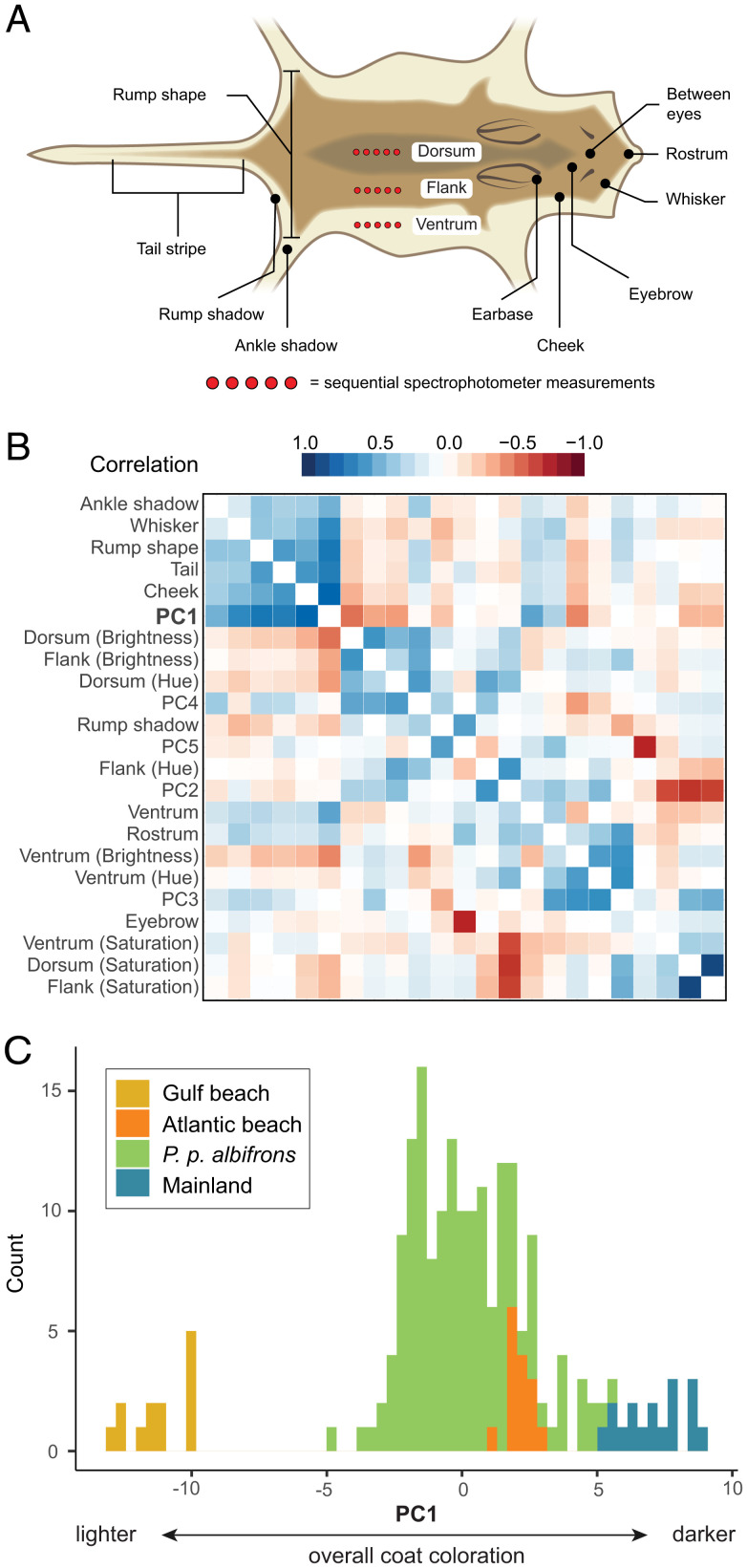
Phenotypic variation in the *P. p. albifrons* population. (*A*) Cartoon showing the traits used to characterize pigment and pattern variation in museum specimens, categorical scores (black), and spectrophotometric measures (red; *Materials and Methods* has details). Image credit: C. Hu, Harvard University, Cambridge, MA. (*B*) Pairwise correlation among pigmentation traits and the first five pPCs in *P. p. albifrons* (*n* = 168). Color indicates direction and strength of the correlation. Invariant traits are not shown. (*C*) Frequency distribution of PC1 scores for *P. p. albifrons* mice as well as representative Gulf (*n* = 13), mainland (*n* = 17), and Atlantic (*n* = 15) mice.

We sampled one mainland population neighboring beach habitat, *P. p. albifrons*, that exhibited a wide range of coat colors—from light and sparsely pigmented coats similar to those of beach mice to the dark and extensively pigmented coats typical of mainland mice. To characterize and quantify this variation, we measured 23 coat-color traits in 168 skin specimens of *P. p. albifrons* (Fig. 2*A*). All traits were related to either the distribution of pigmentation (e.g., tail-stripe length) or intensity of pigment (e.g., dorsal hue, brightness) and are known to vary among beach mouse populations (21). To establish reference points with which to compare the *albifrons* population, we scored the same 23 traits in representative mice from Gulf coast, Atlantic coast, and mainland subspecies (*SI Appendix*, Table S1).

We found that many pigment traits are highly correlated in the *albifrons* population (Fig. 2*B*). A principal components analysis (PCA) shows that six traits—dorsal brightness, tail-stripe length, and the extent of pigmentation on the cheek, rump, whisker, and ankle—heavily load on phenotypic PC1 (pPC1) and that a specimen’s pPC1 score is a strong predictor of overall lightness or darkness (Fig. 2*B* and *SI Appendix*, Fig. S1). Remaining traits also form distinct clusters, but none of these additional clusters encompass as many traits as pPC1 or show the same strength of association with overall coloration (Fig. 2*B*). The highest pPC1 values observed in the population represent the darkest mice, which are similar in appearance (and pPC1 score) to the mainland subspecies *Peromyscus polionotus* (Fig. 2*C*). Additionally, while the lightest *albifrons* individuals are still darker than the geographically proximate beach subspecies *Peromyscus polionotus leucocephalus*—the palest form of the Gulf beach mice—many individuals with intermediate pPC1 scores are comparable with a typical Atlantic beach mouse (e.g., *Peromyscus polionotus niveiventris*) (Fig. 2*C*). Despite this range in coloration that encompasses both beach and mainland phenotypes, none of these pigment traits show a significant association with population structure, not surprising given that the *P. p. albifrons* population has little detectable genetic structure (*SI Appendix*, Fig. S2).

### Association between Pigmentation and a Noncoding Region of *Agouti*.

**Fig. 3.**
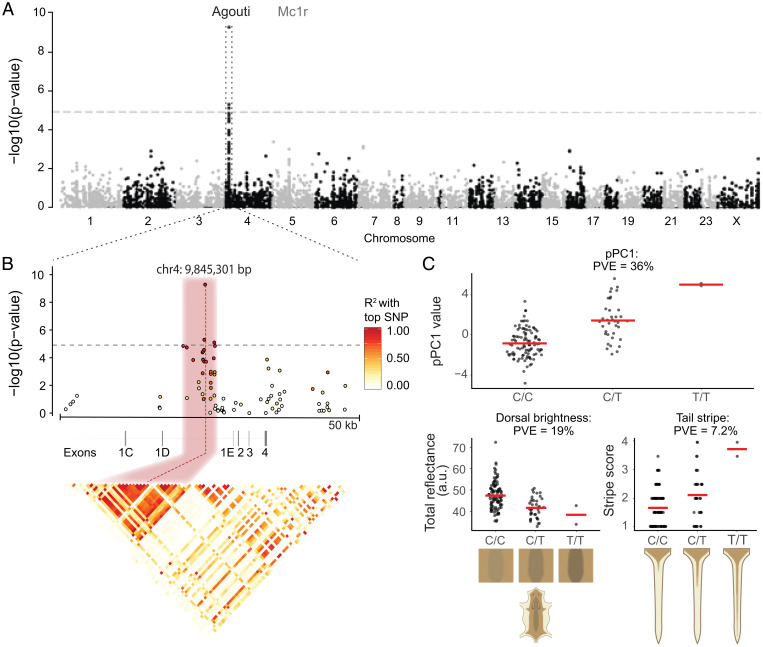
Genotype–phenotype associations in the *Agouti* locus. (*A*) A single peak on chromosome 4 that exceeds the genome-wide significance threshold (dashed line) associates with variation in pPC1. No association is observed in *Mc1r*. (*B*) Zoomed-in view of the *Agouti* association peak. Dots represent variants; color represents the strength of linkage disequilibrium (LD), with the most highly associated SNP variant at chr4: 9,845,301 bp. A single ∼2-kb region (pink) shows high levels of LD. (*Lower*) The correlation matrix displays pairwise LD between all variants in the 50-kb region. (*C*) Distribution of pPC1 scores (*Upper*) and two representative traits (*Lower*) by genotype (C/C, C/T, or T/T) at chr4:9,845,301 bp. Red lines indicate mean trait value by genotype. Cartoons illustrate differences in traits by genotype. PVE, percentage of variance explained.

Capitalizing on the extensive color variation observed within the panmictic *P. p.* *albifrons* population, we performed single-variant association mapping using the sequence-capture data from this population. These data include 6,547 putatively neutral biallelic SNPs from across the genome as well as the genomic regions encompassing two pigmentation genes, *Agouti* and *Mc1r* (190 and 150 kb in length, respectively, including all exons and known regulatory regions). In our scan, we detected a single region associated with pPC1 that exceeded the genome-wide significance threshold (*P* < 1.23 × 10^−5^ corrected for the number of effective tests) (Fig. 3*A*) in the *Agouti* locus. A closer investigation of this region revealed three SNPs significantly associated with pigment variation, spanning 1,756 bp, in strong linkage disequilibrium (mean *r*^2^ = 0.85). A single SNP on chromosome 4 (chr4) at position chr4: 9,845,301 showed a markedly stronger association with pPC1 than the other two (Fig. 3*B*). This SNP is located between two untranslated exons (exons 1D and 1E), is 120 bp upstream of a cluster of Short Interspersed Nuclear Elements (SINE) in reverse orientation relative to the transcription of *Agouti*, and is 5,641 bp upstream of the first coding exon (exon 2). Genotype–phenotype regressions show an additive effect of this locus, which explains 36% of the variance in pPC1, as well as a substantial degree of additive variation in pPC1-correlated traits, such as dorsal brightness (19%) or tail-stripe length (7.2%) (Fig. 3*C*). Together, these data point to a small noncoding region of *Agouti* containing a mutation(s) having a major effect on variation in overall pigmentation in *P. p. albifrons*.

### The Candidate *Agouti* Region Is Capable of Regulatory Activity.

**Fig. 4.**
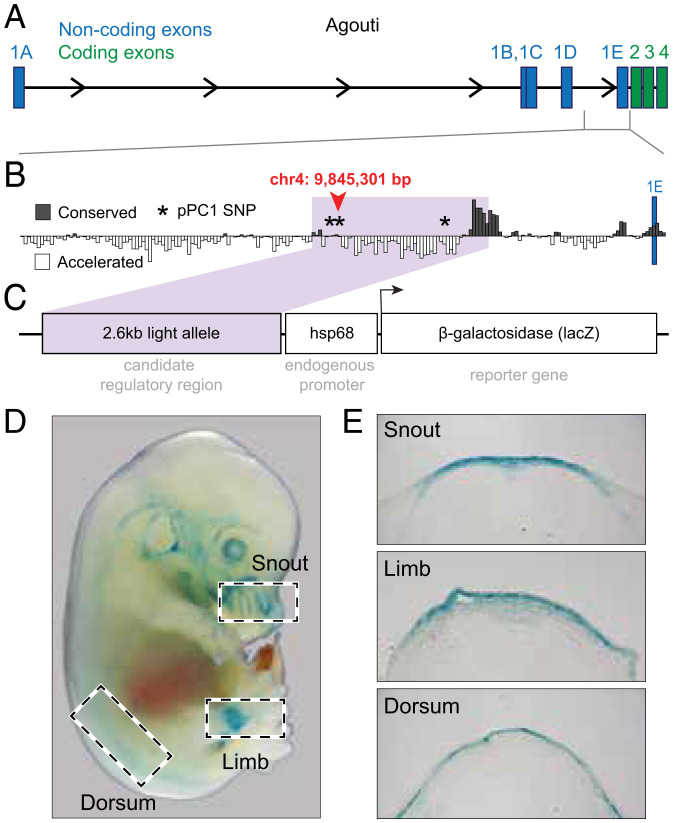
Location, conservation, and activity of the candidate *Agouti* regulatory region. (*A*) Coding structure of the ∼180-kb *Peromyscus Agouti* locus, including noncoding (blue) and coding (green) exons. (*B*) PhyloP sequence conservation among 27 rodent species in the 10 kb encompassing the SNP most highly associated with pPC1 (chr4: 9,845,301); this SNP and two others with a significant pPC1 association are denoted by asterisks. Purple shading highlights the 2.6-kb region cloned into the lacZ reporter plasmid. (*C*) Structure of *lacZ* reporter construct. (*D*) Stage E14.5 transgenic *Mus* (FVB/NJ) embryo stained for *lacZ* expression (blue). (*E*) Three tissue sections showing *lacZ* expression localized to the dermis.

To determine if this ∼2-kb *Agouti* region associated with pPC1 is capable of regulatory activity, we first determined whether the region overlaps with known regulatory elements (Fig. 4*A*). In the homologous region and ±10 kb surrounding sequence in *Mus*, we observe few known regulatory elements, none of which are associated with dermal tissues (*SI Appendix*, Table S2). Moreover, it does not overlap with any previously identified regions associated with pigment variation in other *Peromyscus* species (e.g., ref. 32) (*SI Appendix*, Fig. S3). As sequence conservation can be indicative of conserved molecular function, we next examined sequence similarity across 27 rodents in the 5-kb upstream and downstream of the top-associated SNP (which also includes the two linked variants and SINE elements). Surprisingly, conservation within rodents was minimal, with only a subset of the species—the superfamily Muroidea—showing greater than 50% sequence similarity for the majority of the region (Fig. 4*B* and *SI Appendix*, Fig. S4). These data suggest that if this region has regulatory function, it is likely to have evolved recently.

To assess whether the candidate region of *Agouti* contains functional enhancers, we cloned a 2.6-kb sequence that spans 0.5 kb upstream and 2.1 kb downstream of the most strongly associated variant (i.e., chr4: 9,845,301) and includes the two additional associated variants (chr4: 9,845,152, chr4: 9,846,908) as well as a small downstream region conserved in rodents (Fig. 4*B* and *SI Appendix*, Fig. S5). We then inserted this sequence upstream of a minimal promoter and *lacZ* reporter gene (Fig. 4*C*). Given the currently limited transgenic techniques available for *Peromyscus*, the resulting construct was injected into embryos of *Mus* (strain FVB/NJ), and embryos were collected at embryonic stage (E) 14.5, a time point when *Agouti* expression plays a key role in the establishment of pigment prepatterns in both *Mus* and *Peromyscus* (23). Of the 14 embryos with independent genomic integrations of the *lacZ* construct (verified by PCR), we observed consistent *lacZ* expression in the skin of eight embryos, although expression was spatially variable across embryos (Fig. 4*D* and *SI Appendix*, Fig. S5). Histological analysis showed that *lacZ* was localized to the dermis, corresponding to the known site of endogenous *Agouti* expression during embryonic development (Fig. 4*E*). Together, the results of these experiments suggest that this previously undescribed ∼2-kb noncoding region contains a *cis*-regulatory element (or possibly multiple elements constituting a *cis*-regulatory module) capable of driving *Agouti* dermal expression during embryonic development.

### The Light *Agouti* Allele Shows a Signature of Positive Selection.

**Fig. 5.**
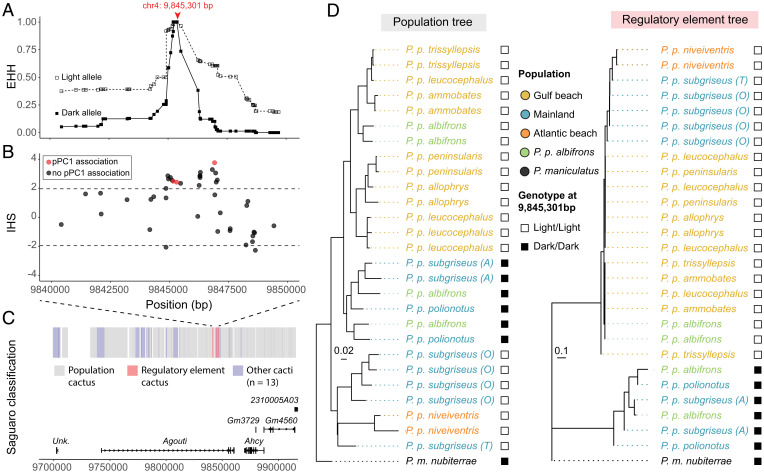
Evolutionary history of the derived light *Agouti* allele. (*A*) EHH decay plot of 10 kb within the *Agouti* locus, showing that the light allele maintains higher levels of homozygosity than the dark allele around the top-associated SNP (chr4: 9,845,301 bp), consistent with a signal of positive selection. (*B*) IHS calculated for the same *Agouti* region. Values of >1.96 indicate statistically significant EHH for the reference allele (*P* < 0.05). (*C*) Saguaro-based classification of local relationships across *Agouti*. Regions that fit to a common single topology (“population cactus”) are shown in gray. Saguaro independently identified a unique topology (“regulatory element cactus”) shown in pink, spanning two neighboring regions of 636 bp (positions 9,841,443 to 9,842,079 bp) and 2,171 bp (9,844,852 to 9,847,023 bp), including the top-associated SNP (chr4: 9,845,301 bp). (*D*) Cactus topologies using *Peromyscus maniculatus* as an outgroup. The population cactus topology closely matches the population tree (shown in Fig. 1*C*), while the regulatory element cactus topology separates individuals homozygous for the light or dark *Agouti* haplotypes, with internal branch lengths suggesting a recent origin of the light *Agouti* allele.

We next tested if there was evidence of natural selection acting on the light-associated allele at this regulatory element, which is found at 86% frequency in the *P. p.* *albifrons* population (*SI Appendix*, Table S3). In the region surrounding the top-associated SNP (chr4: 9,845,301), we found that haplotype homozygosity decays more quickly for the dark allele than for the light allele, a signal consistent with recent positive selection for light pigmentation (Fig. 5*A*). This signal of extended haplotype homozygosity (EHH) is statistically significant, with all 3 candidate SNPs identified in our association analysis (as well as 15 additional SNPs in this region) showing a significantly positive integrated haplotype score (IHS; *P* < 0.05) (Fig. 5*B*). We did not detect a signal of selection at these candidate SNPs in any other population, although low sample sizes and lack of polymorphisms limit our power. Together, these data support the hypothesis that natural selection, most likely favoring light coloration, has led to an increase in light *Agouti* allele frequency in the *P. p. albifrons* population.

### The Light *Agouti* Allele Is Fixed in Both Gulf and Atlantic Beach Mouse Populations.

Given the evidence for nonneutral evolution at this *Agouti* regulatory element, we next aimed to infer whether it exhibits a unique evolutionary history relative to the rest of the genome. Using Saguaro (33), we generated local phylogenies in variably sized genomic windows across the *Agouti* locus for the combined dataset of beach and mainland populations and then compared these phylogenies with the population tree constructed from genome-wide neutral loci. At *Agouti*, we find that much of the locus fits a topology (cactus 6) that coarsely mirrors the population-level phylogeny (Fig. 5*D*). In contrast, one unique topology (cactus 4) is exclusively derived from two small regions that include the top-associated SNP and closely match the regulatory element identified in our mapping experiment (i.e., chr4: 9,841,443 to 9,842,079 and chr4: 9,844,852 to 9,847,023) (Fig. 5*C*). This unique topology clusters all individuals homozygous for *Agouti* light alleles into a single clade with short branch lengths, consistent with a recent origin for this allele, while all dark allele homozygous individuals fall into a clade with longer branch lengths similar to that observed in the population tree (Fig. 5*D*). Therefore, not only does this unsupervised approach identify the same region of *Agouti* as was localized in both the independent association and the selection analyses, but it also points to a single origin for the light *Agouti* allele. These results show that the light *Agouti* allele arose once and is now shared by all Gulf and Atlantic beach mice, even though these lineages are geographically distant and independently colonized their respective beach environments from mainland ancestors.

The light *Agouti* allele, however, occurs in several mainland populations in addition to the *albifrons* population and is likely maintained as standing genetic variation. Consistent with this hypothesis, in the *albifrons* population, we observe elevated levels of sequence divergence (e.g., *D_XY_*) at the *Agouti* regulatory element between light and dark alleles (*SI Appendix*, Fig. S7), suggesting that the light *Agouti* allele may have been introduced to *P. p.* *albifrons* through introgression from another population. This scenario, in which an allele is maintained in several ancestral (mainland) populations and repeatedly selected for in multiple (beach) lineages, suggests that parallel genotypic evolution (sensu ref. 34) has been an important factor in the evolution of light-colored beach mice.

## Discussion

The questions of how adaptation proceeds at the molecular level and how predictable the process is have long been of interest to evolutionary biologists. Debate around, for example, the locus of adaptation (i.e., coding vs. regulatory mutations), the source of adaptive mutations (i.e., de novo mutations vs. preexisting variation), and the repeatability of this process (i.e., do the same or different mutations lead to similar independently evolved traits) has been lively (7, 14, 18, 34–37). Here, we provide insight into these questions from a classic system, first described over a century ago (27): the recent adaptation of two independent lineages of beach mice to white-sand habitat through the evolution of camouflaging color. While the *Agouti* gene has been shown to contribute to the evolution of the Gulf coast beach mouse color pattern through changes affecting both its expression level (22) and spatial domain during embryogenesis (23), the molecular basis of these regulatory changes remained unclear. Furthermore, whether *Agouti* contributed to light pigmentation in the Atlantic coast beach mice remained unknown. Without this information, the question of if the genetic basis of light pigment in the Gulf and Atlantic coast beach mice was the same or different was unknowable. Here, we uncovered a regulatory element in the *Agouti* gene and provided evidence that an allele of this element is associated with lighter pigmentation, which has been selected repeatedly in both lineages of beach mice.

To identify mutation(s) that contribute to changes in pigmentation, we first identified a population (*P. p. albifrons*) that was phenotypically variable, ranging from light beach to dark mainland coat color. While most populations show little variation in pigmentation, this mainland population appears unique, likely because of its geographic proximity (∼25 km) to the beach habitat, its patchwork of light sandy and dark loamy soil, and intermediate level of vegetative cover relative to the open beach and dense mainland habitats. By conducting genetic association mapping in this variable population, we were able to narrow in on a small—approximately 2-kb—noncoding region that is strongly associated with overall pigmentation. This region having a causal effect on pigmentation is bolstered by two additional results. First is its ability to drive expression in the dermis of *Mus* embryos at a developmental stage relevant to the establishment of pigmentation prepatterns. Second, patterns of DNA polymorphism show a strong signature of positive selection in this same small region. Interestingly, this region had not been previously identified as functionally important in *Mus* (26) or *Peromyscus* sp. (32, 38). Moreover, this region is not highly conserved (in rodents), suggesting it may have evolved regulatory function only recently. This regulatory element further supports the observation that *Agouti* regulation is highly modular (26, 32, 38), which could, in turn, explain why *Agouti* expression may be the target of repeated evolutionary tinkering across vertebrates [for example, in rabbits (5), dogs (39), buffalo (40), and birds (41, 42)]—akin to other highly modular genes, such as *Pax6* in vertebrates (43), *Pitx1* in stickleback fish (1, 44), *Ebony* in *Drosophila* (45), and *Asip2b*/*Agrp2* in cichlid fishes (46).

This 2-kb *Agouti* regulatory element likely contains causal mutation(s) that affect pigmentation. In total, there are 11 fixed differences between the light and dark *Agouti* alleles found in beach and mainland mice. While precisely which variants are causal remains unclear, the three of these SNPs that are significantly associated with overall pigmentation (pPC1) in the polymorphic *albifrons* population represent the strongest candidates (*SI Appendix*, Table S5). We also observe several complex insertion/deletions (INDELs) and repetitive elements in the same region, which may themselves affect *Agouti* expression and drive an association signal in linked SNPs. While many of these variants disrupt predicted transcription factor (TF) binding sites identified in *Mus* (*SI Appendix*, Table S4), such predictive approaches have poor specificity, and it is unclear if or when any of these sites may be active. Additionally, because this region is not well conserved even among rodents, results from gene-editing experiments in *Mus*, particularly ones that could result in subtle variation between mutants, may be challenging to interpret. However, future surveys of additional individuals in the admixed *albifrons* population may allow us to pinpoint the causal mutation(s). Similarly, the establishment of dermal cell lines that express *Agouti* in the correct *trans* environment—cell lines that are currently unavailable—could allow us to test the effects of specific mutations or combinations of mutations via cell-based assays.

Nonetheless, with a regulatory element identified, one can now more easily determine the source of this variation. In our survey of *Agouti* variation across both beach and mainland populations, we found that the light *Agouti* allele is fixed in both beach lineages (see below) and also segregating or even fixed in some dark mainland populations. One possibility is that the light *Agouti* allele first arose de novo in one beach lineage and was subsequently introduced through gene flow to the other beach lineage via the mainland population. However, an alternative scenario may be more plausible; the mutation first arose in mainland mice, was maintained as standing variation, and then, was introduced to independent beach lineages via gene flow. First, beach mice are known to have very small populations (31, 47); thus, the opportunity for a new adaptive mutation to arise is low (29). Second, because the beach habitat is relatively young [8 to 10 kya (48)] and colonization of that habitat occurred relatively recently (29), there has been only limited time for a new mutation to arise (much less spread from the beach and across the mainland to the opposite coast via migration). Third, the estimated migration rate from the beach to the mainland is ∼10-fold lower than the reverse (29); this asymmetric migration makes it more likely that substantial genetic variation is being contributed from the mainland rather than to the mainland. Thus, a likely scenario is one in which the light *Agouti* allele originated in a mainland population, was maintained as a standing genetic variation, and then, selected repeatedly in mice that independently colonized the Gulf and Atlantic beaches. This hypothesis is consistent with results from *Mc1r*, in which the age estimate for the emergence of the causal *Mc1r* mutation predates the age of the beach habitat (29).

Interestingly, this scenario was predicted almost a century ago by Francis Sumner (28) based on reports of light-colored mice occurring on the mainland near isolated beach habitats (27). However, while the mainland *P. p.* *albifrons* population represents a likely reservoir of genetic variation, it is unlikely that the light *Agouti* allele originated in this specific population. High sequence divergence in the focal *Agouti* regulatory region between light and dark alleles suggests that the light *Agouti* allele was introduced to the *albifrons* population through introgression from another unsampled (likely mainland) lineage. Regardless of the precise geographic origin of the causal mutation(s), the natural history of the region and the demographic history of these populations together suggest that mainland mice are the likely source of the light *Agouti* allele, which was then used for the rapid and repeated evolution of light-colored beach mice.

This evolutionary scenario then raises the question of how the light *Agouti* allele is maintained in mainland populations, where it may be deleterious. Indeed, previous field experiments demonstrated that models of light mice experienced higher rates of predation than dark models in a dark soil mainland habitat (49). We raise two possible and nonmutually exclusive explanations. First, the light allele may persist in mainland mice because epistasis limits its phenotypic effect; many mainland mice that carry the light *Agouti* allele (even homozygotes) appear to have relatively dark coloration typical of a mainland mouse. In *Peromyscus*, *Agouti* is known to contain multiple mutations that affect pigmentation (32) and to interact with other pigmentation genes (22); therefore, it is possible that epistatic interactions between mutation(s) in the this *Agouti* regulatory element and other mutations in *Agouti* or elsewhere in the genome explain why, in some populations, the light *Agouti* allele has minimal effect on pigmentation (36), thus limiting its visibility to selection. Indeed, previous work in beach mice demonstrated a role for epistasis between *Mc1r* and *Agouti* (22). A second possibility is that the light allele may have a phenotypic effect, even if small, in some mainland populations that persists due to varying selection pressures. In mainland populations, such as *P. p. albifrons*, soil coloration is not uniformly dark but rather, patchy, with sometimes large regions of surprisingly light beach-like substrate. Such mainland areas have light sandy soil due to the geological history of the southeastern United States, which has experienced successive episodes of glacial advance and retreat, depositing light sediments inland and forming sand-dune habitats that remain to this day (50). Thus, the light *Agouti* allele may, at least in some mainland populations, be beneficial, consistent with a signature of positive selection on the light allele in the *P. p.* *albifrons* population. However, the *P. p. albifrons* population also harbors dark *Agouti* alleles, possibly due to spatially variable selection in patchy habitats (e.g., ref. 51) or due to migration from surrounding dark mainland populations (52, 53). Additional sampling through the range of *P. polionotus*, including measurements of soil color, combined with whole-genome resequencing may shed further light on these two hypotheses to explain the prevalence of the light *Agouti* allele in mainland populations.

The distribution of the light allele—segregating in some mainland populations and fixed in both beach mouse populations—is consistent with a scenario in which the same allele was independently selected in the two beach lineages from standing genetic variation. We note, however, that without specific information about the causal mutation(s), we cannot rule out the formal possibility that independent mutations with similar phenotypic effects evolved within the same small regulatory region. However, given the monophyly of the light allele, this would necessitate the unlikely scenario in which independent evolution of causal mutation(s) would have had to occur on the same *Agouti* haplotype. Moreover, given that the Gulf and Atlantic clades are closely related and recently derived from similar mainland ancestors (22), some may argue that this was an ideal scenario for repeated selection on shared ancestral variation (4, 35). Sharing the same *Agouti* light mutation(s) would provide a simple mechanistic explanation for why the Gulf and Atlantic coast beach mice are so similar in pigmentation (19).

Together, these results suggest a scenario in which a *cis*-acting regulatory mutation(s) in *Agouti* likely evolved in the mainland and was independently selected in both the Gulf and Atlantic coast beach mice, contributing to their rapid, parallel evolution. This evolutionary history is in stark contrast with previous results for a second pigmentation gene *Mc1r* (19); a coding change (i.e., a single amino acid mutation) contributes to light coloration in beach mice in Gulf coast beach mice but not Atlantic coast beach mice (21). Thus, together, these two genes demonstrate how—even within a single species and associated with the same adaptive trait—evolution may take very different genetic paths to similar phenotypic ends.

## Materials and Methods

### Specimen and Tissue Collection.

Over two expeditions, in the summer and winter of 2009, we collected 168 *P. p. albifrons* mice from a single population occupying a habitat with patches of both light-colored sand and dark loam–clay soil in Lafayette Creek Wildlife Management Area of Walton County, Florida, ∼25 km inland from the Gulf of Florida (*SI Appendix*, Table S1). Mice were captured overnight using Sherman live traps. Following euthanasia, we sampled liver tissue from each individual and placed the tissues in 95% ethanol until they could be transferred to −80 °C for long-term storage. We also prepared standard museum skins and skeletons. Both tissue and specimens were then accessioned in Harvard University’s Museum of Comparative Zoology (MCZ). In addition to *P. p. albifrons*, we included specimens from 10 distinct beach and mainland locations—representing eight additional *polionotus* subspecies—across the southeastern United States as well as *P. maniculatus nubiterrae* from the northeastern United States as an outgroup (*SI Appendix*, Table S1). Tissues and voucher specimens are accessioned in the Harvard MCZ (*SI Appendix*, Table S5).

### Measurement of Pigment Variation.

We measured 23 pigmentation traits on specimens prepared as flat skins using two approaches: the distribution of pigmentation (e.g., tail-stripe length) or the intensity of pigment (e.g., dorsal hue, brightness). These specific traits were chosen because they are known to vary among beach mouse populations (21, 31); we did not find any new body regions that showed measurable variation within the *albifrons* population. We first scored the extent of pigmentation for 13 body regions, including dorsal, flank, and ventral pigmentation; rump shape and rump shadow; ankle shadow; tail stripe; ear base; eyebrows; cheek; whiskers; rostrum; and between the eyes (Fig. 2*A*). A test set of 10 individuals was scored by two independent researchers, and the methods were refined until their scores were identical. For the full dataset, each trait was scored by a single individual across all specimens to ensure consistency; two researchers scored traits, with each scoring half the traits in all individuals. Second, to measure pigment intensity, we used a FLAME UV-VIS spectrometer with a pulsed xenon light source, a 400-µm reflectance probe, and OceanView software (Ocean Optics) to measure five reflectance spectra from each of three body regions (dorsal stripe, flank, and ventrum). We used a custom R script to obtain brightness, hue, and saturation values in the visible spectrum (400 to 700 nm) with 1-nm bin width using a segment classification approach (54) with formulae as described for CLR v1.05 (55). For all traits, we took five measurements and then, calculated the median value for each body region for each individual. In total, we measured these 23 traits on 168 *P. p. albifrons* specimens as well as representative individuals from the Gulf (*P. p. leucocephalus*, *n* = 13), Atlantic (*P. p. niveiventris*, *n* = 15), and mainland (*P. p. polionotus*, *n* = 17) populations.

### Trait Correlations and Phenotypic PCA.

To test for correlations among traits, we calculated pairwise trait correlations using the cor(., method=”pearson”, use=”complete.obs”) and cor.mtest() functions in base R, correcting for the number of pairwise tests to determine statistical significance (Bonferroni method). To account for trait correlations and to reduce the dimensionality of our dataset, we performed a Principal Component Analysis (PCA) of all pigmentation traits using the FactoMineR v.2.3 (56) and factoextra v.1.0.6 (57) R libraries. More specifically, we first estimated the best number of dimensions for imputing missing data with estim_ncpPCA(., method.cv=”Kfold”), imputed missing data based on the estimated number with imputePCA(., ncp = 5), and then, performed the PCA on the imputed dataset using the PCA() function. Pairwise trait correlations and the phenotypic PCA were based on *P. p. albifrons* individuals only. To compare overall pigmentation (largely captured by pPC1) among populations, pigment scores for individuals from other populations were projected onto the *albifrons* principal component space post hoc using the predict() R function.

### Genome Sequencing and Assembly.

DNA was extracted using standard laboratory procedures from the liver of one female (*P. polionotus subgriseus*; PO stock) obtained from our laboratory colony. By choosing a female individual, we have equal coverage for the autosomes and the X chromosome, but the Y chromosome is not part of the assembly. We prepared libraries with Illumina TruSeq DNA Sample Prep Kit v2 according to the manufacturer’s instructions and performed de novo sequencing on an Illumina HiSeq platform using a combination of short paired-end libraries and longer mate-pair libraries suitable for use with the ALLPATHS-LG genome assembler v.R48559 (58). All libraries were constructed and sequenced at the Broad Institute Sequencing Platform. In total, we generated 240.27 Gb of raw sequence data, representing a total physical coverage of 290× and a sequence coverage of 68×. We assembled these reads using ALLPATHS-LG.

We used ALLMAPS v.0.6.2 (59) in combination with five genetic maps based on interspecific crosses [Restriction site-Associated DNA sequencing (RAD-seq) based (60–63); gene based (64)] to assemble the scaffolds into the pseudochromosomes. DNA sequences corresponding to 182 genes and RAD-seq markers used to build the genetic maps were aligned against the genome using BLAT v.36 × 2 (65). Markers that could not be unambiguously mapped to a single location in the genome were filtered out. A total of 58,922 markers were included in the dataset. During a first iteration, ALLMAPS revealed that a total of 66 scaffolds housed markers associated with more than one linkage group and were likely misassembled. These were subsequently split, and the position of the break points was determined based on the ALLMAPS predictions and the location of discordantly mapped reads. In most cases, these corresponded to assembly gaps. After correcting for these assembly errors, ALLMAPS was run an additional time to generate the pseudochromosomes. Our final assembly includes 531 scaffolds, encompassing 2,575,648,500 bp (97.4% of the total assembled sequence), distributed in 23 autosomes and the X chromosome. The orientation of 461 scaffolds corresponding to 2,566,039,849 bp (97.0% of the total sequence) could be determined due to the presence of more than one marker. We assigned chromosome names based on previous reports from interspecific reciprocal whole-chromosome painting, which allowed us to assign linkage groups with known genes to *Peromyscus* chromosomes (64, 66). The chosen chromosome assignments reflect the standardized *Peromyscus* cytogenetic nomenclature (67).

### Genome Annotation.

We annotated repetitive elements using a combination of RepeatModeler (68) and RepeatMasker v. open-4.0.8 (69) using *Peromyscus*- and rodent-specific repeat libraries. To annotate protein-coding genes, we used a recently developed annotation strategy making use of multiple genome alignments and an existing high-quality annotation set called the Comparative Annotation Toolkit (CAT) v.4c41400 (70). While permitting the finding of newly discovered genes via ab initio gene modeling, this approach allows to identify orthology relationships readily and with high accuracy. We first aligned the oldfield mouse chromosome-level assembly to the assemblies of the laboratory mouse (*Mus musculus*; GRCm38), the rat (*Rattus norvegicus*; Rnor_6.0), the prairie vole (*Microtus ochrogaster*; MicOch1.0), and the prairie deer mouse (*Peromyscus maniculatus bairdii*; Pman2.1.3) using ProgressiveCactus v.0.0 (71, 72). We reasoned that including more species that represent progressive levels of evolutionary divergence would improve the accuracy of the ancestral sequence reconstruction process that takes place during the preparation of the whole-genome alignment. Using CAT, we annotated the oldfield mouse genome using the genome of *M. musculus* (GRCm38/mm10) and the high-quality and well-curated GENCODE VM15 as the reference gene/transcript set as well as extensive transcriptome sequencing datasets for *P. polionotus* corresponding to five tissues (brain, testis, hypothalamus, main olfactory epithelium, and vomeronasal organ) and skin RNA-sequencing (RNA-Seq) data from the prairie deer mouse, *P. maniculatus bairdii*.

To obtain quantitative measures of the completeness of the genome assembly, we used BUSCO v.3.0.2 (73) with BLAST+ v.2.2.28+, HMMER v.3.1b2, and AUGUSTUS v.3.3.2. We used human as species, which specifies the parameters used by AUGUSTUS, and the mammalia and euarchontoglires gene sets for our analyses.

### Population Sequencing, Variant Calling, and Genotype Likelihoods.

For high-coverage WGS of representative beach (*P. p. leucocephalus*) and mainland (*P. p. subgriseus*) populations, we extracted DNA from ∼20 mg of liver tissue and generated sequencing libraries using a PCR-free KAPA HTP kit. Following enzymatic fragmentation, we used size selection to enrich for a 450-bp insert size and ligated Illumina adapters. We sequenced the resulting libraries using 150-bp paired-end sequencing on an Illumina NovaSeq S4 flow cell to achieve 15 to 20× coverage.

For additional Gulf, Atlantic, and mainland populations, we used a sequence-capture strategy aimed at sequencing both putatively neutral loci and the pigmentation genes *Agouti* and *Mc1r*. Specifically, we targeted ∼5,000 1.5-kb noncoding regions randomly distributed across the genome as well as 190- and 150-kb regions flanking the *Agouti* and *Mc1r* loci, respectively (ref. 29 has capture array design details, and ref. 74 has *Agouti* and *Mc1r* sequencing details). This strategy was applied to five Gulf beach mouse subspecies (*Peromyscus p. ammobates*, *Peromyscus p. allophrys*, *Peromyscus p. trisyllepsis*, *Peromyscus p. peninsularis*, *P. p. leucocephalus*), three mainland subspecies (*P. p. polionotus*, *P. p. albifrons*, three populations of *P. p. subgriseus*), and one Atlantic beach subspecies (*P. p. niveiventris*) (*SI Appendix*, Table S1). The availability of both high-quality WGS and sequence-capture data for the *P. p. leucocephalus* and *P. p. subgriseus* subspecies allowed us to verify that the sequence-capture loci accurately represented each population’s genetic diversity.

For both WGS and sequence-capture data, we converted raw fastq files to unmapped bam files using FastqToSam [Picard toolkit v.2.18.4 (75)] and then, marked Illumina adapters using MarkIlluminaAdapters (Picard). Using SamToFastq (Picard), we created interleaved fastq files and clipped adapter sequences. We mapped sequencing reads to the *P. polionotus subgriseus* reference genome (see above) using bwa-mem (76), with –p to indicate interleaved paired-end fastq input and –M to mark short split hits as secondary for compatibility with Picard. We then used MergeBamAlignment (Picard) to merge mapped and unmapped bam files to preserve read group information and sequencing duplicates using MarkDuplicates (Picard), with OPTICAL_DUPLICATE_PIXEL_DISTANCE = 2,500 to account for artifacts generated from the patterned flow cell found in the NovaSeq S4.

We then called variants separately for the WGS and sequence-capture datasets to reduce processing time, as they vary significantly in both coverage and sample number. However, the following variant calling and filtering steps were applied equally to both data types. To begin, we used HaplotypeCaller [GATK v.3.8 (77)] on the aligned bam files with the default heterozygosity prior (-hets = 0.005) and –ERC GVCF to produce per-sample genomic Variant Calling Format (gVCF) files. For the X chromosome, we specified a prior input ploidy based on a comparison of coverage with the autosomes using samtools depth [samtools v.1.10 (78)]. Next, for the WGS data, we generated variant + invariant cohort-level vcfs for each chromosome using GenotypeGVCFs (GATK) with “–max-alternate–alleles 4 -all-sites.” For the sequence-capture data, the “-allsites” parameter was removed, and only variants were reported. These raw cohort-level vcfs were split into INDELs and SNPs with SplitVcfs (Picard) and invariant sites with SelectVariants (GATK). We performed filtering on each set independently, excluding SNPs with Varient Confidence/Quality by Depth (QD) < 2.0, FS > 10.0, Root Mean Square (RMS) Mapping Quality (MQ) < 40.0, MQRankSum < −12.5, ReadPosRankSum < −8.0, or SOR > 3.0 and excluding INDELs with QD < 2.0, Phred-scaled p-value using Fisher's exact test to detect strand bias (FS) > 200.0, ReadPosRankSum < −20.0, or Symmetric Odds Ratio of 2x2 contingency table to detect strand bias/ QUAL – quality score (SOR) > 3.0. We also retained invariant sites with QUAL ≥ 20 using bcftools v.1.11-95 (79). These filtering parameters were based on a combination of GATK recommendations for datasets without truth/training sets and visual inspection of the distributions for each metric. We also set individual genotype calls to missing if the read depth at a given site was less than five. Finally, we combined the sequence-capture dataset with the WGS dataset using vcf-merge [vcftools v.0.1.15 (80)].

### Estimation of Population Structure.

To test for population structure, we ran a gPCA using PCAangsd v.0.973, which is specialized for use with low-coverage, high-throughput sequencing data (81). We used beagle genotype likelihood files for all sequence-capture loci as input and ran the program with default parameters. Using the output covariance matrix, we calculated eigenvalues and eigenvectors with the base R function eigen. We estimated population differentiation (*F_ST_*) for all pairwise population comparisons using the program ANGSD v.0.929-21-g4c6d001 (82). We first calculated the two-population site frequency spectra (2DSFS) using the Site Allele Frequency (SAF) likelihood files generated by ANGSD, running realSFS with default parameters. We then generated the *F_ST_* index for each population pair with the realSFS fst index, supplying each population’s SAF index and the 2DSFS with default parameters. The resulting *F_ST_* index file allowed us to estimate global *F_ST_* as well as *F_ST_* in sliding windows using realSFS fst stats and realSFS fst stats2, respectively.

### Estimation of Population Relationships.

To estimate the relationships among the sampled subspecies, we constructed a population-level tree using the BEAST2 v.2.6.0 application SNAPP, a multispecies coalescent-based tool that uses biallelic markers as input (83–85). Our input data consisted of genome-wide putatively neutral variants sampled in both the sequence-capture and whole-genome datasets (i.e., excluding the *Agouti* and *Mc1r* regions). Briefly, we chose the two highest-coverage individuals representing each population, then retained biallelic SNPs with minor allele frequency (MAF) greater than 0.05, excluded variants that violated Hardy–Weinberg equilibrium (*P* value < 0.001) in four or more populations, and thinned the remaining variants so that none were within 100 bp of each other. The remaining variants were reformatted as a phylip file and converted to the xml format required by SNAPP/BEAST2 using the script snapp_prep.rb (https://raw.githubusercontent.com/mmatschiner/snapp_prep/master/snapp_prep.rb). To specify a starting tree constraint (-s), we ran RAxML v.8.2.12 (86) with ascertainment bias correction (–asc-corr = lewis) on a reduced dataset containing the highest-coverage representative of each subspecies to obtain a maximum likelihood phylogeny. We also specified a node constraint (-c) that the crown divergence of all subspecies, excluding *P. maniculatus nubiterrae* (outgroup), should approximate a normal distribution with a mean of 8.9 kya and an SD of 1.5 kya. These values were taken from SMC++ estimates of the divergence time between mainland (*P. p. subgriseus*) and beach (*P. p. leucocephalus*) subspecies, assuming a generation time of 4 mo (i.e., three generations per year) (*Demographic Inference*). Finally, we sampled 1,000 random variants from the remaining dataset to speed up run times and specified 1 million Markov chain monte carlo (MCMC) iterations. For quality control, we confirmed thorough mixing in the run using Tracer v1.7.1 (87) and visually inspected the trees using DensiTree v.2.2.7 (88). A consensus tree was generated with TreeAnnotator v.2.6.0 (83) using a 10% burn-in and reporting mean node heights.

### Demographic Inference.

The whole-genome, high-density sequencing coverage for one mainland (*P. p. subgriseus*) and one beach (*P. p. leucocephalus)* subspecies allowed us to infer demographic histories with high resolution. Specifically, we used the program SMC++ v1.15.4.dev3+gb53a36d.d20200521 (89) to estimate population divergence times and parameterize population size changes in additional populations. To mask low-quality regions, we followed the SNPable protocol (http://lh3lh3.users.sourceforge.net/snpable.shtml) to identify regions in the assembly with poor mapability using a k-mer size of 150 bp. SNPs that violated Hardy–Weinberg equilibrium (*P* < 0.01) and that had low population coverage (<80% samples genotyped) were also excluded.

We then used the vcf2smc command to create the per-population SMC++ input files, supplying mapability, missingness, and Hardy–Weinberg masks to exclude low-quality regions in the dataset. The “distinguished individual” (DI), a key feature of SMC++, was specified as the highest-coverage sample for each population. We generated two-population input files using the same command and input files but with no specified DI (not applicable to multipopulation analysis). For single-population inference, we used cv with the following parameters: “–folds 4 –timepoints 1e3 5e7 –Nmax 1e8 –spline cubic” and a germline mutation rate of 5.3e-9 (90). We also ran estimate, an earlier version of smc++ cv, with identical parameters for downstream compatibility with population-split inference. We then provided single-population demographic models (as obtained by smc++ estimate) and two-population input files to split to estimate the timing of the mainland (*subgriseus*) and beach (*leucocephalus*) split. To obtain CIs for all the estimates described above, we used a custom script to resample 10-Mb stretches of the genome in the SMC++ input files, thus generating 20 bootstrap replicates per estimate. The above pipeline was rerun with identical parameters on these replicates, and 95% CIs were calculated as mean ± 2 × SE.

### Genome-Wide Association Mapping.

Genotype–phenotype associations were determined using the mixed-model approach implemented in EMMAX v.beta-07Mar2010, accounting for population structure/relatedness by incorporating a Balding–Nichols kinship matrix as a random effect (91). We set the statistical significance threshold at *P* < 0.05 after correcting (Bonferroni method) for the number of effective independent tests obtained with Genetic Type I error calculator v0.2 (92). After excluding samples with more than 50% missing genotypes from these analyses, we were left with *n* = 152 samples. We used both biallelic SNPs and INDELs for association mapping but excluded markers with >50% missing data, with an MAF of <0.05, or deviating from Hardy–Weinberg equilibrium (*P* < 0.001). We generated Manhattan plots and Quantile-Quantile (QQ) plots using the qqman v.0.1.4 (93) and snpStats v.1.32.0 (94) R libraries, respectively. Using plink v1.90b6.15, we calculated pairwise linkage disequilibrium (*r*^2^) among SNPs in the focal region (flags: –chr chr4 –from-bp 9820301 –to-bp 9870301 –r2 –ld-window-r2 0 –ld-window 1000). Next, we estimated the proportion of variance explained for a given SNP (assuming Hardy–Weinberg equilibrium) using genotype–phenotype regressions.

### Sequence Conservation.

To evaluate the nucleotide sequence conservation level of the *Agouti* locus in *P. polionotus*, including the candidate regulatory region, we downloaded all available orthologous rodent *Agouti* sequences from National Center for Biotechnology Information (NCBI; accessed 7 September 2020) using esearch (-db gene -query “ortholog_gene_434[group] AND rodents[orgn]”) in combination with esummary and extract from EDirect v.13.8. Next, we manually added 15 kb to each of the start and end coordinates (or the maximum number of base pairs if hitting a scaffold end) using a custom awk script and retrieved the corresponding nucleotide sequences with efetch. The sequence of *Nannospalax galili* was removed due to a lack of available flanking sequence. Finally, we determined sequence conservation between *P. polionotus* and the remaining 26 rodent species using mVISTA [accessed 8 September 2020 (95)] and phyloP v.1.4 (96). For phyloP, we provided a phylogeny of the 27 species based on data from TimeTree [accessed 15 June 2021 (97)].

### Regulatory Database Queries

To determine if the candidate regulatory region of *Agouti* contains any known regulatory elements or TF binding sites, we downloaded both phastCons60way conserved elements and ORegAnno regulatory elements from the University of California Santa Cruz (UCSC) genome browser in *M. musculus* mm10 coordinates (http://hgdownload.cse.ucsc.edu/goldenpath/mm10/database/). Elements from each database were converted to *P. polionotus* genomic coordinates using UCSC’s liftOver v.358 (65) and a custom chain file, with the parameters “-multiple -minMatch = 0.70.”

We obtained ENSEMBL regulatory features using the R package biomaRt v.2.38.0 (98, 99). The mm39 regulatory feature dataset was retrieved with the function useDataset(), with the parameters “dataset=mmusculus_regulatory_feature,” mart=“ENSEMBL_MART_FUNCGEN,” and getBM() used to retrieve entries from the broader *Agouti* region using the extended *Agouti* coordinates (2:154785921:155055915) for the mm39 assembly. We directly converted coordinates in mm39 to mm10 assembly coordinates using liftOver with default parameters and the UCSC mm39toMm10 chain file (https://hgdownload.soe.ucsc.edu/goldenPath/mm39/liftOver/mm39ToMm10.over.chain.gz); then, we converted mm10 coordinates to *P. polionotus* coordinates using the same approach described above.

### LacZ Reporter Assay.

To determine if the candidate region was capable of regulatory activity, we assessed whether it could drive expression of the *lacZ* reporter gene in the skin of developing mouse embryos (strain FVB/NJ). To identify the most appropriate sequence length for this experiment, we specified boundaries that encompassed the three pPC1-significant SNPs, the unique local topology regions identified by Saguaro (*Local Tree Inference with* Saguaro), and the tract of relatively high sequence conservation at the 3′ end of the association and Saguaro regions, resulting in a total sequence length of 2.6 kb (Fig. 4*B*). While *lacZ* experiments are particularly useful for verifying that a regulatory locus is active, comparisons between alleles of the same locus (e.g., light and dark alleles) can be challenging due to the noise associated with random genomic integration of the construct. Therefore, presented with two alternative haplotypes in this region—“light” and “dark”—we decided to use the light haplotype for these experiments under the assumption that the light allele was less likely to contain mutations reducing element activity (i.e., high Agouti expression is generally associated with light pigmentation).

We used the *lacZ* expression vector hsp68lacZ (a gift from T. Capellini, Harvard University, Cambridge, MA; Addgene no. 37843). The light haplotype sequence file and hsp68lacZ vector were provided to Taconic Biosciences, which synthesized and cloned the sequence upstream of the hsp68 minimal promoter followed by pronuclear microinjection, collection of E14.5 embryos, genotyping, and *lacZ* staining. Stained embryos were photographed, embedded in Optimal Cutting Temperature compound (OCT), cryosectioned, and imaged in house.

### TF Binding Site Prediction.

To determine if variation in the regulatory element could be modifying relevant TF binding sites, we examined motif differences at variant positions across the region. Specifically, we obtained all polymorphic sites in the regulatory element (chr4: 9,844,852 to 9,847,500 bp) with MAF > 0.05 in the *P. p. albifrons* population. We extracted the region 15 bp upstream and downstream of each variant (∼30-bp sequence) and used vcf-consensus (vcftools) to create an alternate sequence incorporating the variant. For each reference and alternate sequence, we used CiiiDER v.0.9 (100) to predict TF binding sites, providing the database of 251 *M. musculus* CORE TF position weight matrices available on JASPAR [downloaded 20 October 2021 (101)].

### Haplotype Homozygosity Tests.

To test for evidence of nonneutral evolution in patterns of nucleotide variation, we calculated haplotype statistics. We first ran fastPHASE v1.4.8 to create phased variant calls (102). We converted the input vcf for all individual genotypes at *Agouti*, *Mc1r*, and the sequence-capture loci to the fastPHASE format with vcf2fastPHASE.pl (https://github.com/lstevison/vcf-conversion-tools); then, we ran fastPHASE with the following parameters: -T20 -H50 -F. We next converted the phased output back to the vcf format with fastPHASE2VCF.pl.

Using the R package rehh v.3.1.0, we ran a series of haplotype-based tests to scan for signatures of positive selection on the light and dark haplotypes (103). For each population, we converted vcfs to an rehh-compatible file with a custom script (hap2rehh.py) using the *P. polionotus subgriseus* reference genome to polarize alleles. We then converted these files to haplohh objects with data2haplohh and computed extended haplotype homozygosity (EHH) statistics with scan_hh, with the parameters “discard_integration_at_border = FALSE, maxgap = 2000” to accommodate the sequence-capture dataset. We then ran ihh2ihs to calculate IHSs using an MAF filter of 0.05 and default allele frequency bin sizes of 0.025.

### Local Tree Inference with Saguaro.

As a complementary approach to test for evidence of selection within *Agouti*, we used the hidden Markov model (HMM)–based software Saguaro v0.1 to build local phylogenies from sequence data (33). As input, we used variant calls from the sequence-capture dataset and the *Agouti* and *Mc1r* extended loci and filtered out variants with MAF < 0.025. To reduce computational complexity and help with downstream interpretation, we reduced the sample size to include only the two highest-coverage representatives of each population. In the case of *albifrons*, we included two individuals homozygous for the dark allele and two for the light allele (as determined by their genotype at SNP chr4: 9,845,301 bp). We then used VCF2HMMFeature to transform the variant calls to a Saguaro-compatible input format. We ran Saguaro for 15 iterations with default parameters. We transformed the resulting topologies to phylip files with Saguaro2Phylip and obtained HMM transitions from the LocalTrees.out file.

### Sequence Diversity of the *Agouti* Regulatory Allele.

To characterize sequence diversity in the *Agouti* regulatory region, we calculated *F_ST_*, *D_XY_*, and $\theta_{\pi }$ using scikit-allel v.1.3.2 (104). Specifically, we classified *albifrons* individuals by genotype at the top associated *Agouti* SNP (chr4: 9,845,301): light/light (*n* = 107), light/dark (*n* = 37), and dark/dark (*n* = 2). In 100-bp nonoverlapping windows, we calculated Hudson’s *F_ST_*, *D_XY_*, and $\theta_{\pi }$ using the functions allel.windowed_hudson_fst(), allel.windowed_divergence(), and allel.windowed_diversity(), respectively. Given the sparse nature of the sequence-capture dataset, we restricted these calculations to 100-bp windows where at least 70% of *albifrons* individuals had five or more mapped reads. We then used these data to determine 95, 99, and 99.5% genomic outliers for *D_XY_* and *F_ST_*.

## Supplementary Material

Supplementary File

## Data Availability

The *P. polionotus subgriseus* reference genome is archived in the Whole Genome Sequences BioProject Database (accession no. PRJNA494229) (105). Whole-genome and target-capture sequence data are archived in the NCBI Sequence Read Archive BioProject Database (accession no. PRJNA838595) (106). Scripts and phenotypic data are available in GitHub (https://github.com/twooldridge/Agouti_enhancer_paper) (107). Genome assembly data have been deposited in NCBI (accession no. GCA_003704135.2) (108). All other data are included in the article and/or *SI Appendix*.
