# Supplementary material for: An enhancer of Agouti contributes to parallel evolution of cryptically colored beach mice
Source: Proc Natl Acad Sci U S A. 2022 Jul 1;119(27):e2202862119. doi: 10.1073/pnas.2202862119 (PMC9271204; doi:10.1073/pnas.2202862119)
Supplement: Supplementary File [file pnas.2202862119.sapp.pdf]

Supplementary Information for

An enhancer of *Agouti* contributes to parallel evolution of cryptically colored beach mice

T. Brock Wooldridge, Andreas F. Kautt, Jean-Marc Lassance, Sade McFadden, Vera S. Domingues, Ricardo Mallarino, Hopi E. Hoekstra

Correspondence to: hoekstra@oeb.harvard.edu

This file includes:

Tables S1 to S5
Figures S1 to S7
SI References

Table S1. Sampling information. List of species, subspecies, and populations included in this study. "Collector/Publications" points to information for the precise sampling method, time, and place. Primary collector initials are as follows: EPK = Evan Kingsley, NLB = Nicole Bedford, VSD = Vera Domingues. "Sequencing strategy" refers to either WGS = whole genome sequencing (highlighted in grey) or Seqcapture = targeted sequence capture array.

Taxonomy	Collectors/ Publications	Latitude	Longitude	No. samples	Sequencing strategy
P. polionotus ammobates	Mullen *et al.* 2009 (*1*)	30.229978	-87.814703	15	Seqcapture
P. polionotus allophrys	Mullen *et al.* 2009 (*1*)	30.077795	-85.647814	11	Seqcapture
P. polionotus leucocephalus	NLB	30.397536	-86.729057	15	WGS
P. polionotus leucocephalus	Mullen et al. 2009 (*1*)	30.397536	-86.729057	20	Seqcapture
P. maniculatus nubiterrae	EPK	40.33	-79.27	1	Seqcapture
P. polionotus trisyllepsis	Mullen *et al.* 2009 (*1*)	30.29371	-87.463557	5	Seqcapture
P. polionotus subgriseus	VSD	29.1828333	-81.795	15	WGS
P. polionotus albifrons	VSD	30.5411	-86.075717	168	Seqcapture
P. polionotus polionotus	Domingues *et al.* 2012 (*2*)	31.995717	-85.082967	6	Seqcapture
P. polionotus subgriseus (A)	Domingues *et al.* 2012 (*2*)	30.814577	-84.954529	5	Seqcapture
P. polionotus subgriseus (T)	Domingues *et al.* 2012 (*2*)	31.6459	-84.225	5	Seqcapture
P. polionotus subgriseus (O)	Domingues *et al.* 2012 (*2*)	29.207927	-81.740378	5	Seqcapture
P. polionotus peninsularis	Mullen *et al.* 2009 (*1*)	29.957198	-85.462412	19	Seqcapture
P. polionotus niveiventris	Steiner *et al.* 2009 (*3*)	27.923493	-80.488186	4	Seqcapture

Table S2. Regulatory elements in *Agouti*. *P. polionotus*-based coordinates (HU_Ppol1.3.3) of regulatory features lifted over from the ENSEMBL *Mus musculus* genome (GRCm39). Features found ~10 kb upstream and downstream of the 2-kb candidate region are shown.

P. polionotus chromosome	*P. polionotus* start (bp)	*P. polionotus* end (bp)	*M. musculus* chromosome	*M. musculus* start (bp)	*M. musculus* end (bp)	ENSEMBL ID	ENSEMBL element type
chr4	9834661	9834859	chr2	154872801	154873001	ENSMUSR00000830170	Predicted enhancer
chr4	9842998	9843607	chr2	154880275	154880693	ENSMUSR00000636464	Transcription factor binding
chr4	9845488	9845887	chr2	154881887	154882302	ENSMUSR00000830171	Open chromatin region
chr4	9850467	9851000	chr2	154887001	154887600	ENSMUSR00000830172	Predicted enhancer
chr4	9852071	9852243	chr2	154888801	154889000	ENSMUSR00000830173	CTCF Binding Site
chr4	9855030	9857063	chr2	154892000	154894001	ENSMUSR00000636468	Predicted promoter

Table S3. Population-level frequency of light allele. Each species is provided with its source location. Frequency of the light allele is calculated as the proportion of haplotypes in a population that have the light-associated allele at chr4:9,845,301 bp.

Species	Location	Frequency of light *Agouti* allele
P. p. allophrys	Gulf Coast Beach	1
P. p. ammobates	Gulf Coast Beach	1
P. p. leucocephalus	Gulf Coast Beach	1
P. p. trisyllepsis	Gulf Coast Beach	1
P. p. peninsularis	Gulf Coast Beach	1
P. p. niveiventris	Atlantic Coast Beach	1
P. m. nubiterrae	Mainland (Outgroup)	0
P. p. albifrons	Mainland	0.86
P. p. polionotus	Mainland	0
P. p. subgriseus (A)	Mainland	0
P. p. subgriseus (O)	Mainland	1
P. p. subgriseus (T)	Mainland	1

Table S4. Transcription factor binding sites overlapping variant positions in the regulatory element of *P. p. albifrons*. "Position" includes only sites on chr4 in the tested regulatory element (9,844,852 bp – 9,847,500 bp) that are variant in *P. p. albifrons* with a minor allele frequency (MAF) > 0.05. Both "Reference allele" and "Alternate allele" are relative to the *P. p. subgriseus* reference genome (no relation to light or dark haplotypes). "Reference-specific TFs" and "Alternate-specific TFs" refer to predicted TF binding sites from JASPAR (*4*) (see Methods) that are intact in the Reference allele and Alternate allele, respectively. pPC1-associated SNPs are highlighted in gray.

Position	Reference allele	Alternate allele	Alternate allele frequency	Reference-specific TFs	Alternate-specific TFs
9844852	G	A	0.0648148	Foxo3;Neurog1	Arid5a
9844869	GC	G	0.168712	Zfp335	Rhox11
9844930	G	A	0.171975	--	Rhox11
9844954	A	G	0.159091	--	Mitf
9844955	G	GTC	0.155844	--	Mitf
9845040	T	A	0.154762	--	Nkx3-2
9845116	C	T	0.140741	Dlx5	Nkx2-5
9845136	G	GA	0.142336	Foxn1	--
9845152	G	A	0.150735	Arnt::Hif1a;Hes1	Pax2
9845301	C	T	0.140411	--	Nkx3-2
9845470	C	T	0.127049	--	--
9846196	C	T	0.208333	Gfi1b;Klf4	Barhl1;Bcl11b
9846254	G	A	0.0620915	--	Atoh1;Bhlha15;Msgn1;Myc; Npas2;Twist2
9846286	A	G	0.123333	Atoh1;Bhlha15;Bhlhe40;Fos::Jun;Foxj2;Foxo1;Foxo3;Mitf;Myc;Npas2;Pax2;Twist2	--
9846292	T	C	0.125	Atf3;Atoh1;Bhlha15;Bhlhe40;Fos::Jun;Foxj2;Foxo1;Foxo3;Jun;Mitf;Myc;Nkx3-2;Npas2;Twist2	Nr2e1
9846293	G	A	0.121622	Atf3;Atoh1;Bhlha15;Bhlhe40;Fos::Jun;Foxj2;Foxo1;Foxo3;Jun;Mitf;Myc;Nkx3-2;Npas2;Twist2	Nr2e1
9846308	T	C	0.127586	Atoh1	Hes2
9846815	G	A	0.143357	--	Nkx2-5
9846908	A	G	0.171975	Cebpa;Mafb	Elf5;Hand1::Tcf3;Hic1
9846919	C	T	0.0796178	--	--
9846954	T	G	0.102564	--	--
9846977	C	T	0.115385	--	Barhl1;Neurog1;Nkx2-5
9847023	T	G	0.153846	Elf5;Gata1	Rfx6
9847042	T	G	0.730263	--	Hand1::Tcf3
9847150	A	G	0.788591	Barhl1;Nkx2-5	Myb;Stat6;Tcf3

Table S5. Museum accession information for all samples included in study. "Sample ID" refers to ID used by authors in this study, while "Collection ID" refers to the ID used by the institution where samples are accessioned. "Collection" refers to either MCZ = Harvard Museum of Comparative Zoology, or Lab = internal Hoekstra Lab specimen collection (samples available upon request).

Sample ID	Species	Collection	Collection ID
01_NB_F_EPK04	*P. m. nubiterrae*	not accessioned	NA
VSD142	*P. p. albifrons*	MCZ	68140
VSD143	*P. p. albifrons*	MCZ	68141
VSD195	*P. p. albifrons*	MCZ	68192
VSD196	*P. p. albifrons*	MCZ	68193
VSD197	*P. p. albifrons*	MCZ	68194
VSD198	*P. p. albifrons*	MCZ	68195
VSD199	*P. p. albifrons*	MCZ	68196
VSD200	*P. p. albifrons*	MCZ	68197
VSD201	*P. p. albifrons*	MCZ	68198
VSD202	*P. p. albifrons*	MCZ	68199
VSD203	*P. p. albifrons*	MCZ	68200
VSD204	*P. p. albifrons*	MCZ	68201
VSD205	*P. p. albifrons*	MCZ	68202
VSD207	*P. p. albifrons*	MCZ	68204
VSD208	*P. p. albifrons*	MCZ	68205
VSD209	*P. p. albifrons*	MCZ	68206
VSD210	*P. p. albifrons*	MCZ	68207
VSD211	*P. p. albifrons*	MCZ	68208
VSD212	*P. p. albifrons*	MCZ	68209
VSD214	*P. p. albifrons*	MCZ	68211
VSD215	*P. p. albifrons*	MCZ	68212
VSD216	*P. p. albifrons*	MCZ	68213
VSD217	*P. p. albifrons*	MCZ	68214
VSD218	*P. p. albifrons*	MCZ	68215
VSD219	*P. p. albifrons*	MCZ	68216
VSD220	*P. p. albifrons*	MCZ	68217
VSD221	*P. p. albifrons*	MCZ	68218
VSD222	*P. p. albifrons*	MCZ	68219
VSD223	*P. p. albifrons*	MCZ	68220
VSD224	*P. p. albifrons*	MCZ	68221
VSD225	*P. p. albifrons*	MCZ	68222
VSD226	*P. p. albifrons*	MCZ	68223
VSD227	*P. p. albifrons*	MCZ	68224
VSD228	*P. p. albifrons*	MCZ	68225
VSD229	*P. p. albifrons*	MCZ	68226
VSD230	*P. p. albifrons*	MCZ	68227
VSD231	*P. p. albifrons*	MCZ	68228
VSD233	*P. p. albifrons*	MCZ	68230
VSD234	*P. p. albifrons*	MCZ	68231
VSD235	*P. p. albifrons*	MCZ	68232
VSD236	*P. p. albifrons*	MCZ	68233
VSD237	*P. p. albifrons*	MCZ	68234
VSD238	*P. p. albifrons*	MCZ	68235
VSD239	*P. p. albifrons*	MCZ	68236
VSD240	*P. p. albifrons*	MCZ	68237
VSD241	*P. p. albifrons*	MCZ	68238
VSD242	*P. p. albifrons*	MCZ	68239
VSD243	*P. p. albifrons*	MCZ	68240
VSD244	*P. p. albifrons*	MCZ	68241
VSD245	*P. p. albifrons*	MCZ	68242
VSD246	*P. p. albifrons*	MCZ	68243
VSD247	*P. p. albifrons*	MCZ	68244
VSD248	*P. p. albifrons*	MCZ	68245
VSD249	*P. p. albifrons*	MCZ	68246
VSD250	*P. p. albifrons*	MCZ	68247
VSD251	*P. p. albifrons*	MCZ	68248
VSD252	*P. p. albifrons*	MCZ	68249
VSD253	*P. p. albifrons*	MCZ	68250
VSD254	*P. p. albifrons*	MCZ	68251
VSD256	*P. p. albifrons*	MCZ	68253

VSD257	*P. p. albifrons*	MCZ	68254
VSD259	*P. p. albifrons*	MCZ	68256
VSD260	*P. p. albifrons*	MCZ	68257
VSD261	*P. p. albifrons*	MCZ	68258
VSD262	*P. p. albifrons*	MCZ	68259
VSD263	*P. p. albifrons*	MCZ	68260
VSD264	*P. p. albifrons*	MCZ	68261
VSD265	*P. p. albifrons*	MCZ	68262
VSD266	*P. p. albifrons*	MCZ	68263
VSD267	*P. p. albifrons*	MCZ	68264
VSD268	*P. p. albifrons*	MCZ	68265
VSD269	*P. p. albifrons*	MCZ	68266
VSD270	*P. p. albifrons*	MCZ	68267
VSD271	*P. p. albifrons*	MCZ	68268
VSD272	*P. p. albifrons*	MCZ	68269
VSD273	*P. p. albifrons*	MCZ	68270
VSD274	*P. p. albifrons*	MCZ	68271
VSD275	*P. p. albifrons*	MCZ	68272
VSD276	*P. p. albifrons*	MCZ	68273
VSD278	*P. p. albifrons*	MCZ	68275
VSD279	*P. p. albifrons*	MCZ	68276
VSD280	*P. p. albifrons*	MCZ	68277
VSD281	*P. p. albifrons*	MCZ	68278
VSD282	*P. p. albifrons*	MCZ	68279
VSD283	*P. p. albifrons*	MCZ	68280
VSD284	*P. p. albifrons*	MCZ	68281
VSD285	*P. p. albifrons*	MCZ	68282
VSD286	*P. p. albifrons*	MCZ	68283
VSD287	*P. p. albifrons*	MCZ	68284
VSD288	*P. p. albifrons*	MCZ	68285
VSD289	*P. p. albifrons*	MCZ	68286
VSD292	*P. p. albifrons*	MCZ	68289
VSD293	*P. p. albifrons*	MCZ	68290
VSD294	*P. p. albifrons*	MCZ	68291
VSD295	*P. p. albifrons*	MCZ	68292
VSD296	*P. p. albifrons*	MCZ	68293
VSD298	*P. p. albifrons*	MCZ	68295
VSD299	*P. p. albifrons*	MCZ	68296
VSD307	*P. p. albifrons*	MCZ	68304
VSD308	*P. p. albifrons*	MCZ	68305
VSD309	*P. p. albifrons*	MCZ	68306
VSD310	*P. p. albifrons*	MCZ	68307
VSD311	*P. p. albifrons*	MCZ	68308
VSD312	*P. p. albifrons*	MCZ	68309
VSD313	*P. p. albifrons*	MCZ	68310
VSD314	*P. p. albifrons*	MCZ	68311
VSD315	*P. p. albifrons*	MCZ	68312
VSD316	*P. p. albifrons*	MCZ	68313
VSD317	*P. p. albifrons*	MCZ	68314
VSD318	*P. p. albifrons*	MCZ	68315
VSD319	*P. p. albifrons*	MCZ	68316
VSD320	*P. p. albifrons*	MCZ	68317
VSD321	*P. p. albifrons*	MCZ	68318
VSD322	*P. p. albifrons*	MCZ	68319
VSD323	*P. p. albifrons*	MCZ	68320
VSD324	*P. p. albifrons*	MCZ	68321
VSD331	*P. p. albifrons*	MCZ	68328
VSD332	*P. p. albifrons*	MCZ	68329
VSD333	*P. p. albifrons*	MCZ	68330
VSD334	*P. p. albifrons*	MCZ	68331
VSD335	*P. p. albifrons*	MCZ	68332
VSD336	*P. p. albifrons*	MCZ	68333
VSD337	*P. p. albifrons*	MCZ	68334
VSD338	*P. p. albifrons*	MCZ	68335
VSD339	*P. p. albifrons*	MCZ	68336
VSD340	*P. p. albifrons*	MCZ	68337
VSD341	*P. p. albifrons*	MCZ	68338
VSD342	*P. p. albifrons*	MCZ	68339
VSD343	*P. p. albifrons*	MCZ	68340
VSD344	*P. p. albifrons*	MCZ	68341

VSD345	*P. p. albifrons*	MCZ	68342
VSD346	*P. p. albifrons*	MCZ	68343
VSD347	*P. p. albifrons*	MCZ	68344
VSD348	*P. p. albifrons*	MCZ	68345
VSD349	*P. p. albifrons*	MCZ	68346
VSD350	*P. p. albifrons*	MCZ	68347
VSD351	*P. p. albifrons*	MCZ	68348
VSD352	*P. p. albifrons*	MCZ	68349
VSD353	*P. p. albifrons*	MCZ	68350
VSD354	*P. p. albifrons*	MCZ	68351
VSD355	*P. p. albifrons*	MCZ	68352
VSD356	*P. p. albifrons*	MCZ	68353
VSD357	*P. p. albifrons*	MCZ	68354
VSD358	*P. p. albifrons*	MCZ	68355
VSD63	*P. p. albifrons*	MCZ	68061
VSD65	*P. p. albifrons*	MCZ	68063
VSD66	*P. p. albifrons*	MCZ	68064
VSD67	*P. p. albifrons*	MCZ	68065
VSD69	*P. p. albifrons*	MCZ	68067
VSD70	*P. p. albifrons*	MCZ	68068
VSD71	*P. p. albifrons*	MCZ	68069
VSD72	*P. p. albifrons*	MCZ	68070
VSD73	*P. p. albifrons*	MCZ	68071
VSD74	*P. p. albifrons*	MCZ	68072
VSD75	*P. p. albifrons*	MCZ	68073
VSD76	*P. p. albifrons*	MCZ	68074
VSD77	*P. p. albifrons*	MCZ	68075
VSD78	*P. p. albifrons*	MCZ	68076
VSD79	*P. p. albifrons*	MCZ	68077
VSD80	*P. p. albifrons*	MCZ	68078
VSD81	*P. p. albifrons*	MCZ	68079
VSD82	*P. p. albifrons*	MCZ	68080
VSD83	*P. p. albifrons*	MCZ	68081
VSD84	*P. p. albifrons*	MCZ	68082
VSD85	*P. p. albifrons*	MCZ	68083
VSD86	*P. p. albifrons*	MCZ	68084
VSD87	*P. p. albifrons*	MCZ	68085
VSD88	*P. p. albifrons*	MCZ	68086
CBM2000	*P. p. allophrys*	Lab	
CBM245	*P. p. allophrys*	Lab	
CBM259	*P. p. allophrys*	Lab	
CBM264	*P. p. allophrys*	Lab	
CBM310	*P. p. allophrys*	Lab	
CBM415	*P. p. allophrys*	Lab	
CBM6000	*P. p. allophrys*	Lab	
CBM973	*P. p. allophrys*	Lab	
MCZ65946	*P. p. allophrys*	MCZ	65946
MCZ65947	*P. p. allophrys*	MCZ	65947
allophrys_MCZ65945	*P. p. allophrys*	MCZ	65945
ABM101	*P. p. ammobates*	Lab	
ABM105	*P. p. ammobates*	Lab	
ABM2298	*P. p. ammobates*	Lab	
ABM2618	*P. p. ammobates*	Lab	
ABM2639	*P. p. ammobates*	Lab	
ABM2766	*P. p. ammobates*	Lab	
ABM3	*P. p. ammobates*	Lab	
ABM4240	*P. p. ammobates*	Lab	
ABM4637	*P. p. ammobates*	Lab	
ABM473	*P. p. ammobates*	Lab	
ABM5	*P. p. ammobates*	Lab	
ABM501	*P. p. ammobates*	Lab	
ABM6	*P. p. ammobates*	Lab	
MCZ65932	*P. p. ammobates*	MCZ	65932
ammobates_MCZ65930	*P. p. ammobates*	MCZ	65930
4434	*P. p. leucocephalus*	MCZ	4434
69677	*P. p. leucocephalus*	MCZ	69677
69679	*P. p. leucocephalus*	MCZ	69679
69686	*P. p. leucocephalus*	MCZ	69686
7817	*P. p. leucocephalus*	MCZ	7817
7819	*P. p. leucocephalus*	MCZ	7819

7820	*P. p. leucocephalus*	MCZ	7820
7824	*P. p. leucocephalus*	MCZ	7824
7827	*P. p. leucocephalus*	MCZ	7827
7828	*P. p. leucocephalus*	MCZ	7828
7830	*P. p. leucocephalus*	MCZ	7830
7831	*P. p. leucocephalus*	MCZ	7831
7832	*P. p. leucocephalus*	MCZ	7832
7835	*P. p. leucocephalus*	MCZ	7835
7838	*P. p. leucocephalus*	MCZ	7838
SRIBM110	*P. p. leucocephalus*	Lab	
SRIBM1400	*P. p. leucocephalus*	Lab	
SRIBM160	*P. p. leucocephalus*	Lab	
SRIBM222	*P. p. leucocephalus*	Lab	
SRIBM333	*P. p. leucocephalus*	Lab	
SRIBM334	*P. p. leucocephalus*	Lab	
SRIBM335	*P. p. leucocephalus*	Lab	
SRIBM336	*P. p. leucocephalus*	Lab	
SRIBM337	*P. p. leucocephalus*	Lab	
SRIBM338	*P. p. leucocephalus*	Lab	
SRIBM339	*P. p. leucocephalus*	Lab	
SRIBM430	*P. p. leucocephalus*	Lab	
SRIBM440	*P. p. leucocephalus*	Lab	
SRIBM520	*P. p. leucocephalus*	Lab	
SRIBM531	*P. p. leucocephalus*	Lab	
SRIBM600	*P. p. leucocephalus*	Lab	
SRIBM688	*P. p. leucocephalus*	Lab	
SRIBM734	*P. p. leucocephalus*	Lab	
SRIBM822	*P. p. leucocephalus*	Lab	
SRIBM920	*P. p. leucocephalus*	Lab	
MCZ66104	*P. p. niveiventris*	MCZ	66104
MCZ66105	*P. p. niveiventris*	MCZ	66105
MCZ66107	*P. p. niveiventris*	MCZ	66107
mcz66106	*P. p. niveiventris*	MCZ	66106
SABM11	*P. p. peninsularis*	Lab	
SABM12	*P. p. peninsularis*	Lab	
SABM137	*P. p. peninsularis*	Lab	
SABM142	*P. p. peninsularis*	Lab	
SABM145	*P. p. peninsularis*	Lab	
SABM146	*P. p. peninsularis*	Lab	
SABM159	*P. p. peninsularis*	Lab	
SABM175	*P. p. peninsularis*	Lab	
SABM20	*P. p. peninsularis*	Lab	
SABM21	*P. p. peninsularis*	Lab	
SABM22	*P. p. peninsularis*	Lab	
SABM24	*P. p. peninsularis*	Lab	
SABM248	*P. p. peninsularis*	Lab	
SABM5	*P. p. peninsularis*	Lab	
SABMI174	*P. p. peninsularis*	Lab	
SABM_ECI154	*P. p. peninsularis*	Lab	
peninsularis_MCZ65948	*P. p. peninsularis*	MCZ	65948
sabm247	*P. p. peninsularis*	Lab	
sabm3	*P. p. peninsularis*	Lab	
JNW49	*P. p. polionotus*	MCZ	64653
JNW50	*P. p. polionotus*	MCZ	64654
VSD176	*P. p. polionotus*	MCZ	68174
VSD182	*P. p. polionotus*	MCZ	68180
VSD186	*P. p. polionotus*	MCZ	68184
VSD188	*P. p. polionotus*	MCZ	68186
VSD2	*P. p. subgriseus (A)*	MCZ	68002
VSD4	*P. p. subgriseus (A)*	MCZ	68004
VSD5	*P. p. subgriseus (A)*	MCZ	68005
VSD7	*P. p. subgriseus (A)*	MCZ	68007
VSD9	*P. p. subgriseus (A)*	MCZ	68009
68144	*P. p. subgriseus (O)*	MCZ	68144
68146	*P. p. subgriseus (O)*	MCZ	68146
68147	*P. p. subgriseus (O)*	MCZ	68147
68148	*P. p. subgriseus (O)*	MCZ	68148
68149	*P. p. subgriseus (O)*	MCZ	68149
68150	*P. p. subgriseus (O)*	MCZ	68150
68151	*P. p. subgriseus (O)*	MCZ	68151

68152	*P. p. subgriseus (O)*	MCZ	68152
68153	*P. p. subgriseus (O)*	MCZ	68153
68154	*P. p. subgriseus (O)*	MCZ	68154
68159	*P. p. subgriseus (O)*	MCZ	68159
68160	*P. p. subgriseus (O)*	MCZ	68160
68161	*P. p. subgriseus (O)*	MCZ	68161
68162	*P. p. subgriseus (O)*	MCZ	68162
68163	*P. p. subgriseus (O)*	MCZ	68163
VSD148	*P. p. subgriseus (O)*	MCZ	68146
VSD150	*P. p. subgriseus (O)*	MCZ	68148
VSD154	*P. p. subgriseus (O)*	MCZ	68152
VSD156	*P. p. subgriseus (O)*	MCZ	68154
VSD163	*P. p. subgriseus (O)*	MCZ	68161
VSD118	*P. p. subgriseus (T)*	MCZ	68116
VSD120	*P. p. subgriseus (T)*	MCZ	68118
VSD122	*P. p. subgriseus (T)*	MCZ	68120
VSD123	*P. p. subgriseus* (T)	MCZ	68121
VSD127	*P. p. subgriseus* (T)	MCZ	68125
PKBM104	*P. p. trisyllepsis*	Lab	
PKBM1042	*P. p. trisyllepsis*	Lab	
PKBM1077	*P. p. trisyllepsis*	Lab	
PKBM1112	*P. p. trisyllepsis*	Lab	
PKBM145	*P. p. trisyllepsis*	Lab	

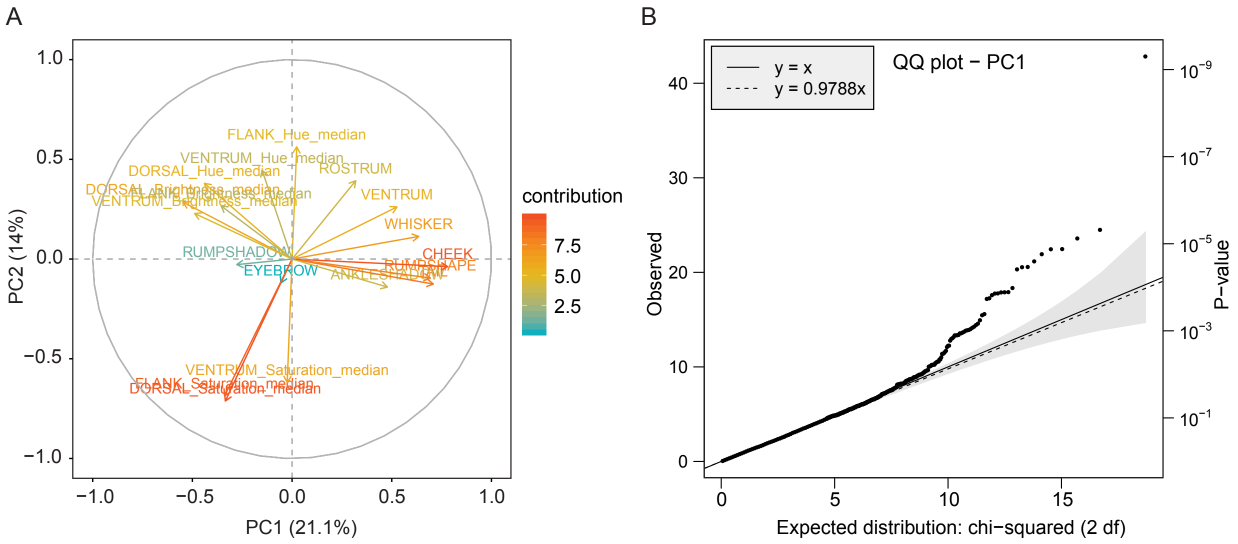

Figure S1. Pigment trait loadings on phenotypic Principal Component Analysis (pPCA). **A**. pPCA biplot shows contributions of pigment traits to the first two phenotypic PCs. Percentage values in parentheses correspond to the percent variance explained by each PC. **B**. Quantile-quantile plot of empirical vs. expected GWAS p-value distributions, indicating no signs of overdispersion or abnormal behavior.

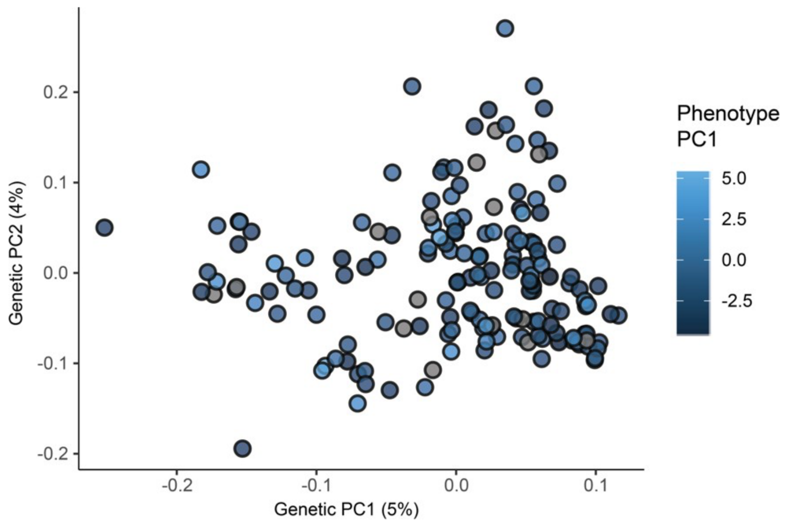

Figure S2. Genetic principal components analysis of the *P. p. albifrons* population. Each dot represents an individual (N=168). Color approximates phenotypic PC1 value. Percentage value on each axis corresponds to the percent variance explained by each genetic PC.

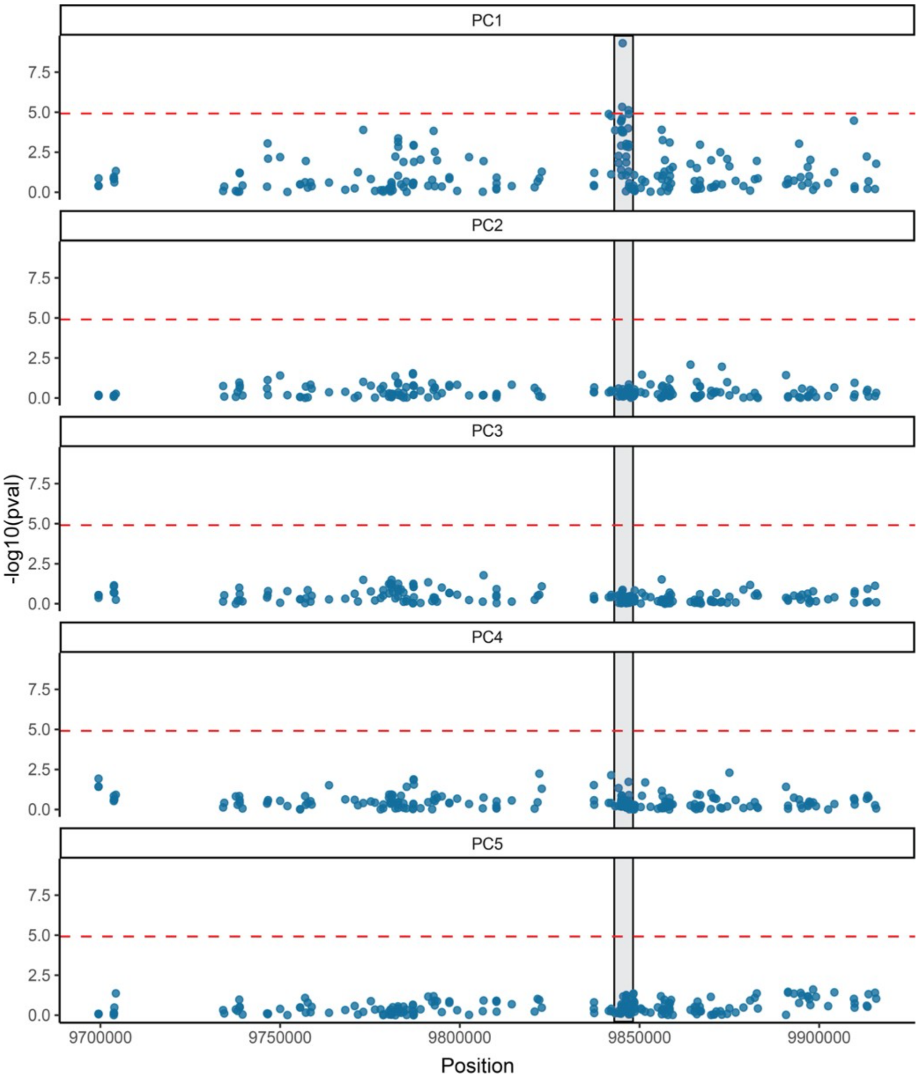

Figure S3. Manhattan plots showing association between phenotype and variation across the *Agouti* locus. Data for phenotypic PCs (pPCs) 1 to 5 are shown. Dashed red lines indicate genome-wide significant threshold, corrected for number of independent tests (see Methods). Gray bar denotes boundaries of peak association found for pPC1. No other pPCs show a significant association with variants in *Agouti.*

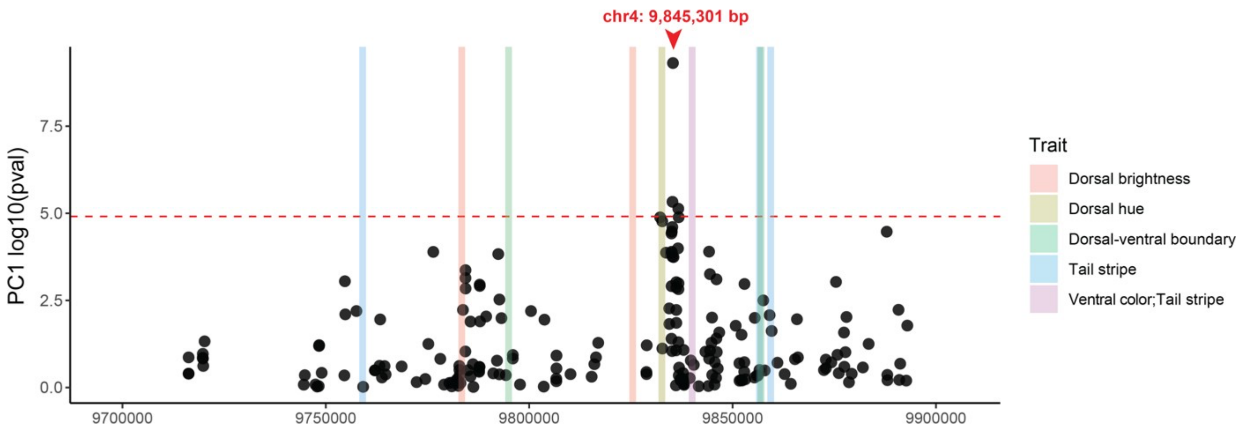

Figure S4. Location of the identified regulatory region in relation to previously implicated regions. The top-associated SNP (chr4:9,845,301) is shown in red. Vertical bars indicate regions of *Agouti* that are significantly associated with pigmentation traits in *P. maniculatus*, from (*5*).

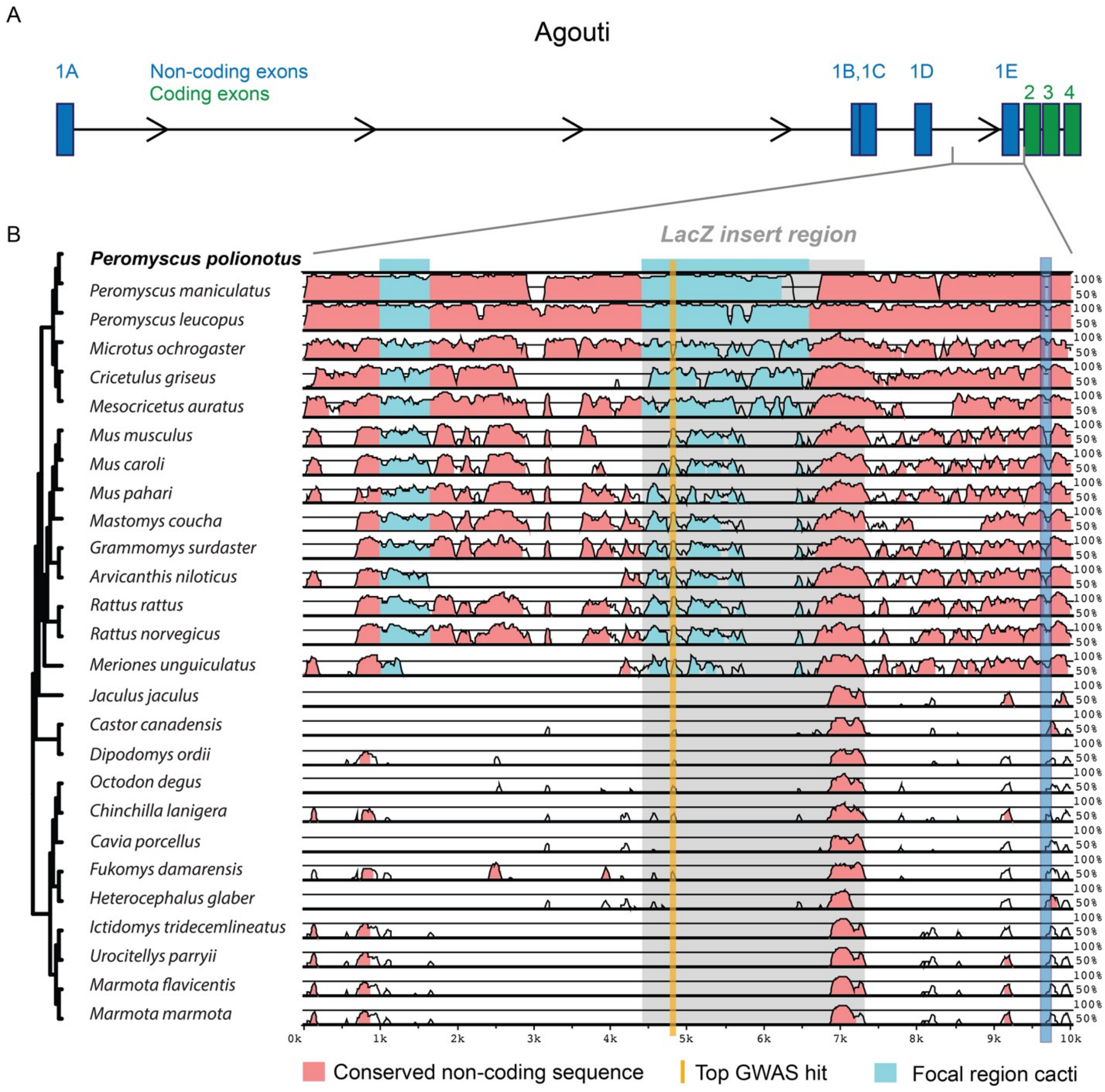

Figure S5. Sequence conservation in *Agouti* among 27 rodent species. 10-kb region encompassing the SNP with the strongest association to pPC1 (chr4:9,845,301) denoted in gold. Conserved regions are shown in pink. 'Focal region cacti' (light blue) indicate the regions identified by Saguaro (*6*) (see Methods; Fig. 5) with a unique topology relative to the rest of the *Agouti* locus. The 2.6-kb region used in the *lacZ* reporter assay (grey) includes the cacti region (light blue), the top associated SNP (gold) and a conserved region (pink). One non-coding exon, 1E, is shown as a landmark (dark blue).

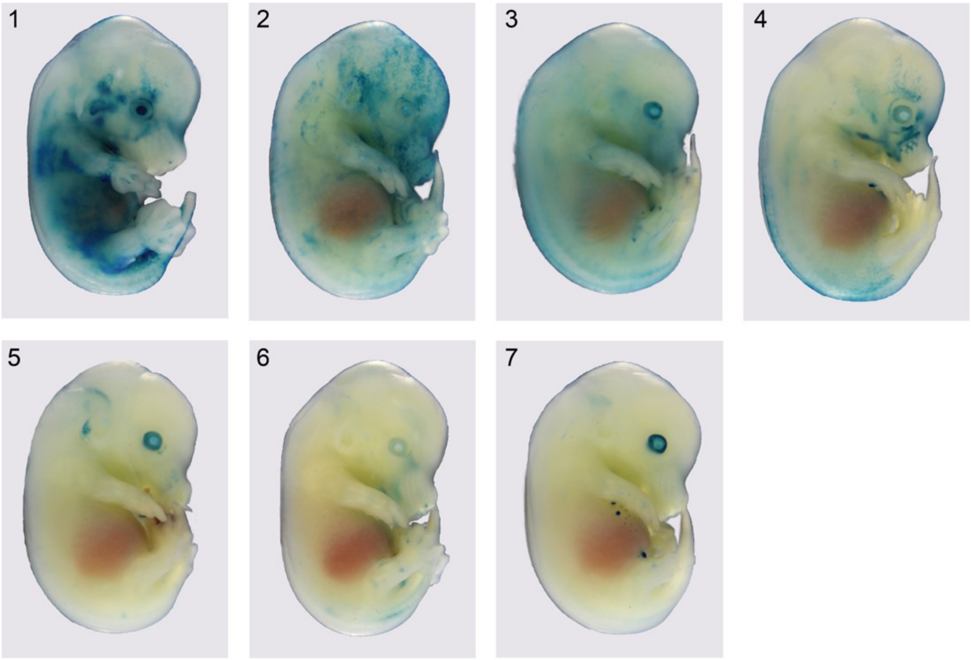

Figure S6. Transgenic embryos with visible skin *lacZ* expression produced by pronuclear injection of the candidate region-lacZ vector. All embryos are at stage E14.5, PCR-positive for the *lacZ* vector, and each represents an independent genomic integration event. Blue staining shows *lacZ* expression and regulatory element activity.

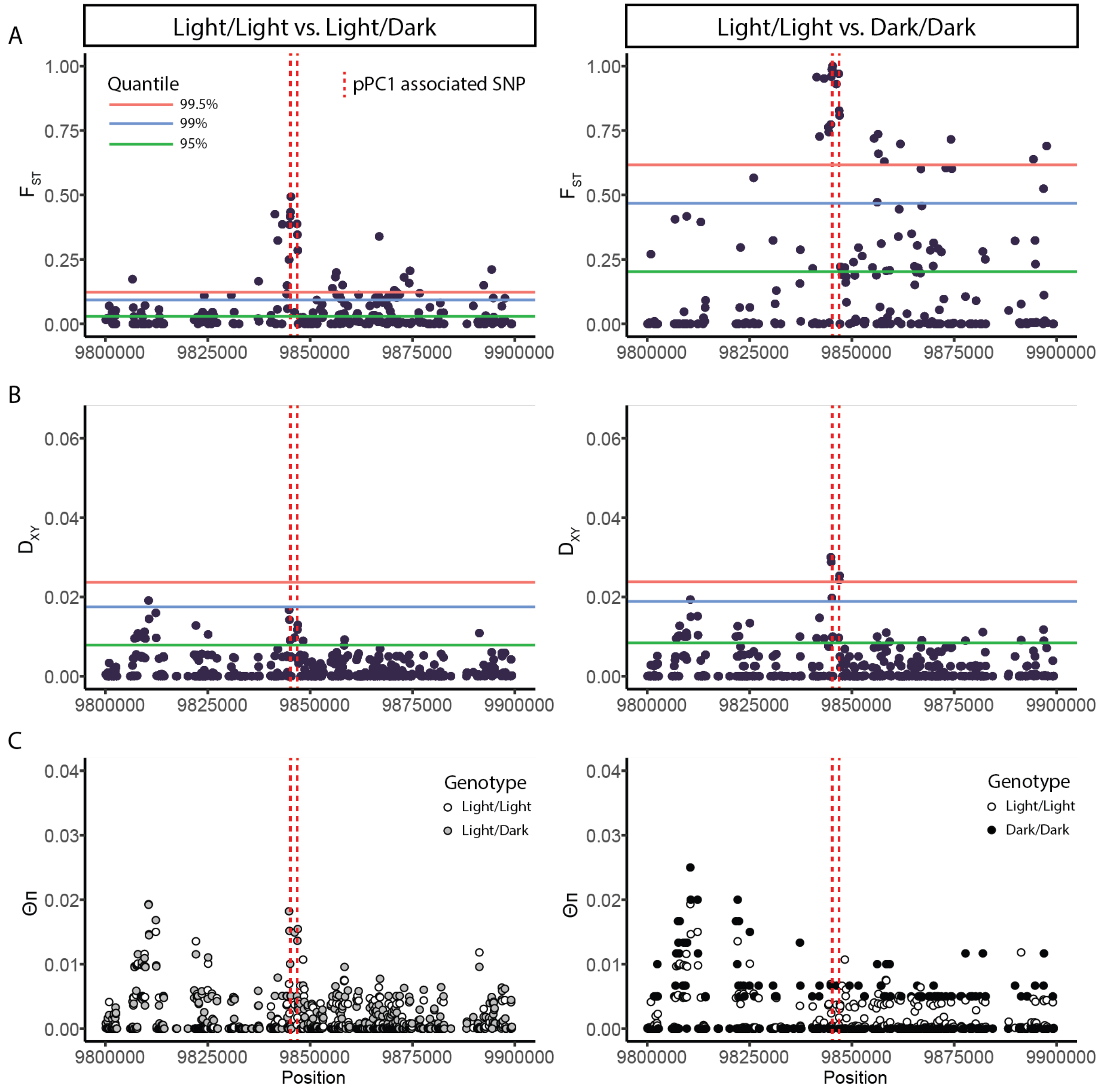

Figure S7. Sequence divergence among *Agouti* regulatory allele genotypes in the *P. p. albifrons* population. Left panels show comparisons between *albifrons* individuals homozygous for the light *Agouti* allele (light/light; n = 107) and heterozygous light/dark (n = 37) at the top associated SNP (chr4:9,845,301). Right panels compare light/light (n = 107) and dark/dark (n = 2) genotypes. Despite unbalanced sample sizes, signals of elevated F_{ST} and D_{XY} at the focal regulatory element (denoted by vertical red dotted lines corresponding to the 3 SNPs associated with phenotypic PC1) are seen in both comparisons. All points correspond to 100-bp non-overlapping windows. **A.** Hudson's F_{ST}. **B.** D_{XY}. **C.** θ!, split by genotype group.

References

1. Mullen LM, Vignieri SN, Gore JA, Hoekstra HE. 2009. Adaptive basis of geographic variation: genetic, phenotypic and environmental differences among beach mouse populations. *Proc Royal Soc B* **276**:3809–3818.
2. Domingues VS, Poh Y, Peterson BK, Pennings PS, Jensen JD, Hoekstra HE. 2012. Evidence of adaptation from ancestral variation in young populations of beach mice. *Evolution* **66**:3209–3223.
3. Steiner CC, Rompler H, Boettger LM, Schoneberg T, Hoekstra HE. 2009. The genetic basis of phenotypic convergence in beach mice: similar pigment patterns but different genes. *Mol Biol Evol* **26**:35–45.
4. Fornes O, Castro-Mondragon JA, Khan A, van der Lee R, Zhang X, Richmond PA, Modi BP, Correard S, Gheorghe M, Baranašić D, Santana-Garcia W, Tan G, Chèneby J, Ballester B, Parcy F, Sandelin A, Lenhard B, Wasserman WW, Mathelier A. 2020. JASPAR 2020: update of the open-access database of transcription factor binding profiles. *Nucleic Acids Res* **48**:D87–D92.
5. Linnen CR, Poh Y-P, Peterson BK, Barrett RDH, Larson JG, Jensen JD, Hoekstra HE. 2013. Adaptive evolution of multiple traits through multiple mutations at a single gene. *Science* **339**:1312–1316.
6. Zamani N, Russell P, Lantz H, Hoeppner MP, Meadows JR, Vijay N, Mauceli E, di Palma F, Lindblad-Toh K, Jern P, Grabherr MG. 2013. Unsupervised genome-wide recognition of local relationship patterns. *BMC Genomics* **14**:347.
